# Supplementary material for: Burkholderia pseudomallei BipD modulates host mitophagy to evade killing
Source: Nat Commun. 2024 Jun 4;15:4740. doi: 10.1038/s41467-024-48824-x (PMC11150414; doi:10.1038/s41467-024-48824-x)
Supplement: Supplementary file 1 — Supplementary Information [file 41467_2024_48824_MOESM1_ESM.pdf]

Supplementary Information

Title: *Burkholderia pseudomallei* BipD modulates host mitophagy to evade killing

Authors: Dongqi Nan#, Chenglong Rao#, Zhiheng Tang#, Wenbo Yang#, Pan Wu, Jiangao Chen, Yupei Xia, Jingmin Yan, Wenzheng Liu, Ziyuan Zhang, Zhiqiang Hu, Hai Chen, Yaling Liao, Xuhu Mao*, Xiaoyun Liu*, Quanming Zou*, Qian Li*

#Equally Contributing Authors

***Correspondence**: Qian Li, E-mail: liqianjane@163.com

Quanming Zou, E-mail: qmzou@tmmu.edu.cn

Xiaoyun Liu, E-mail: xiaoyun.liu@bjmu.edu.cn

Xuhu Mao, E-mail: maoxh2012@hotmail.com

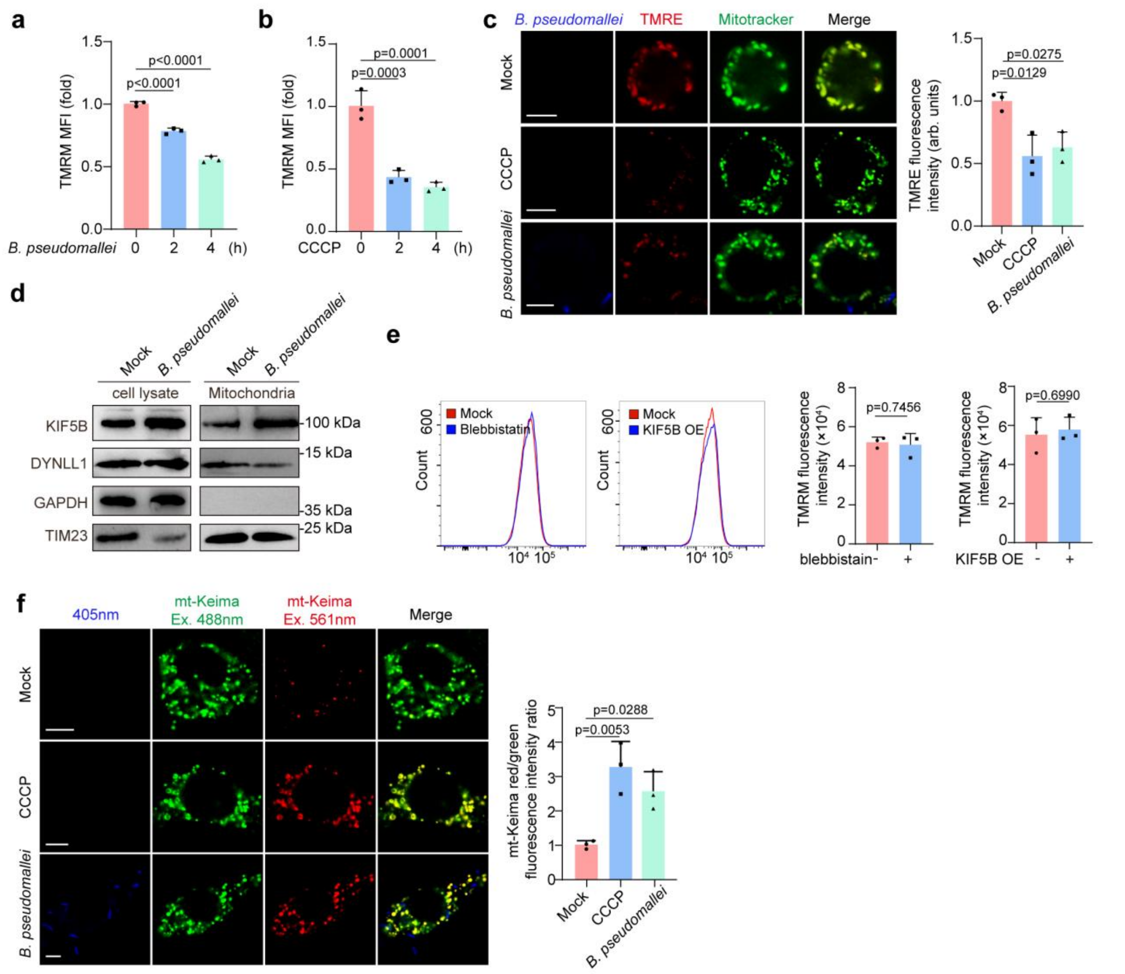

Supplementary Figure 1. *B. pseudomallei* infection induced host mitophagy not mitocytosis

(**a** and **b**) Flow cytometry analysis of mitochondrial membrane potential (MMP, Δψm) in RAW264.7 cells by TMRM staining. Cells were treated with 10 μM CCCP or infected with *B. pseudomallei* at the MOI of 10 for indicated time points.

(**c**) Immunofluorescence staining with TMRE (100 nM, red) and Mitotracker Green (75 nM, green) was performed in RAW264.7 cells. Scale bar, 5 μm. Data were from 3 independent experiments with 30 cells in each group.

(**d**) Western blot analysis of purified mitochondrial fractions from cells after *B. pseudomallei* infection in RAW264.7 cells. Mock stands for uninfected group.

(**e**) FACS analysis of the effect of myosin II inhibitor blebbistatin or KIF5B overexpression (promoting migrasome formation) on MMP in RAW264.7 cells after *B. pseudomallei* infection. TMRM fluorescence intensity was quantified from 3 independent experiments.

(**f**) Representative confocal immunofluorescence microscopy images of mitochondria in RAW264.7 cells transfected with adenoviruses encoding mt-Keima after CCCP treatment or *B. pseudomallei* infection. Both CCCP treatment and *B. pseudomallei* infection elevated the presence of mitochondria in mature autolysosomes, indicating a strong mitophagy signal compared to the controls. Cells were observed at 488 nm (green) or 561 nm (red) light, respectively. Scale bar, 5 μm. The mt-Keima red/green fluorescence intensity ratio was quantified from 3 independent experiments. The above data indicate means ± SD for three independent trials. One-way ANOVA

followed by Tukey post hoc test (**a**, **b**, **c**, **f**) and two-tailed Student's *t* test (**e**) were used for data analysis. n = 3 in each group (**a**, **b**, **c**, **e**, **f**). Source data were provided as a Source Data file.

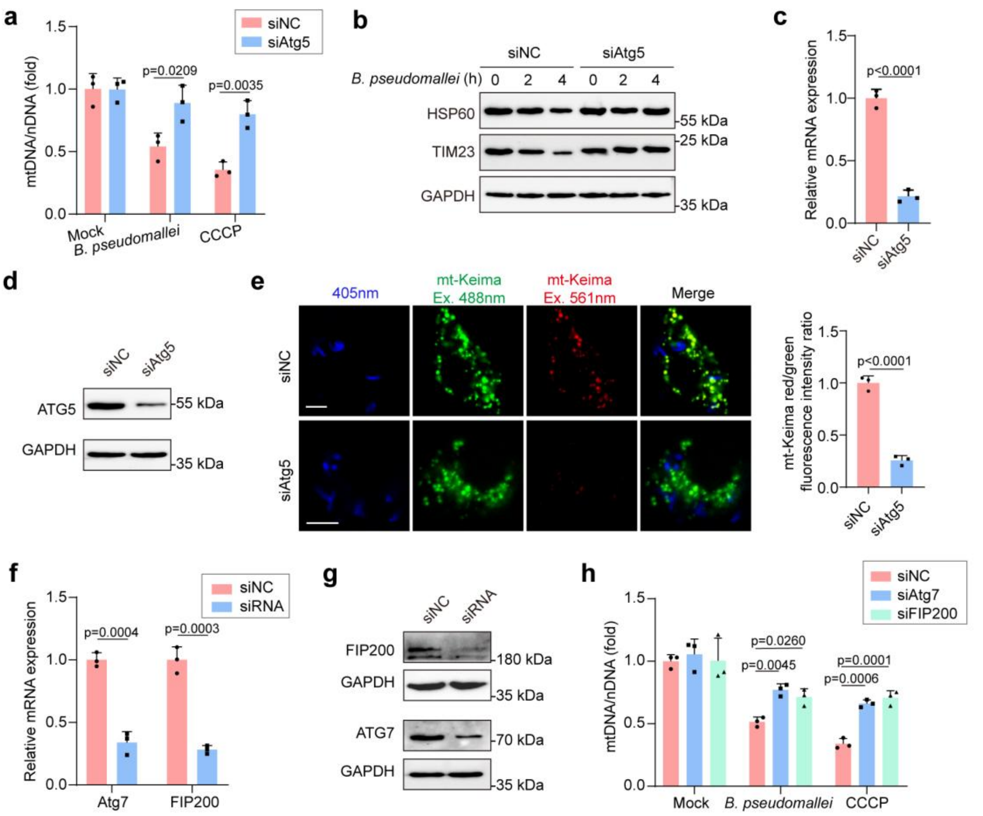

Supplementary Figure 2. Canonical autophagy is required for *B. pseudomallei*-induced mitophagy.

(**a**) Quantification of mtDNA/nDNA levels in siNC (siRNA control) or siAtg5 treated RAW264.7 cells by qPCR analysis. Cells were infected with *B. pseudomallei* or CCCP (10 μM).

(**b**) Western blot analysis of mitochondrial proteins HSP60 and TIM23 in RAW264.7 cells infected with *B. pseudomallei* at an MOI of 10 for the indicated time points.

(**c** and **d**) Knockdown efficiency of siAtg5 was measured.

(**e**) Representative immunofluorescence images of mitochondria to lysosomes using mt-Keima assay. Quantification of mt-Keima fluorescence intensity of the siNC or siAtg5 treated RAW264.7 cells infected with *B. pseudomallei*. Scale bar, 5 μm.

(**f** and **g**) Knockdown efficiency of siAtg7 and siFIP200 was detected, respectively.

(**h**) mtDNA/nDNA ratio was measured in RAW264.7 cells that were treated with siNC or siRNA targeting *Atg5* or *FIP200*. Cells were treated as in (**a**). The above data indicate means ± SD for three independent trials. One-way ANOVA followed by Tukey post hoc test (**h**) and two-tailed Student's *t* test (**a**, **c**, **f**) were used for data analysis. n = 3 in each group (**a**, **c**, **f**, **h**). Source data were provided as a Source Data file.

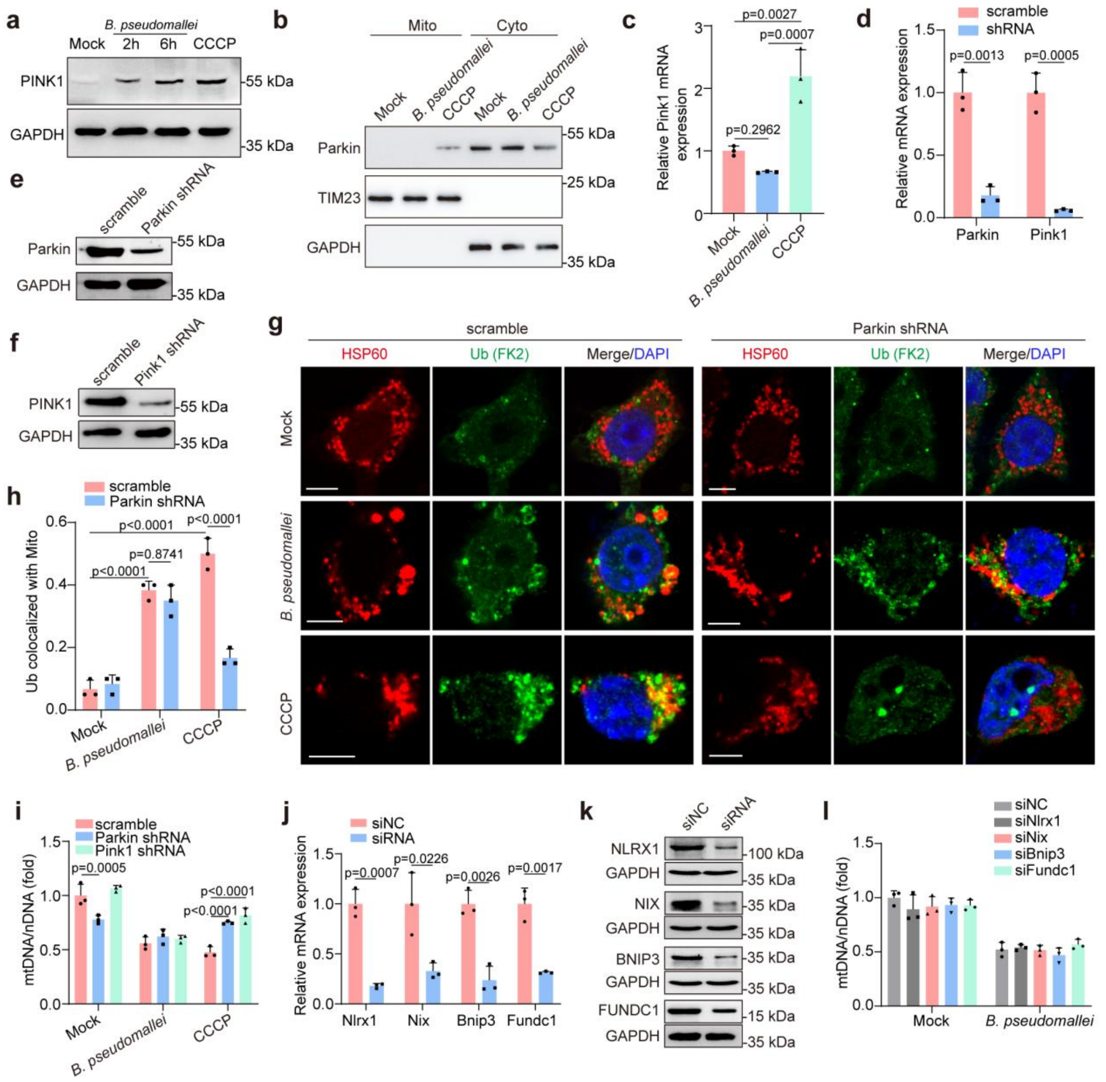

Supplementary Figure 3. *B. pseudomallei* infection may induce host mitophagy in a Ub-dependent pathway

(**a**) Determined of PINK1 level in RAW264.7 cells with *B. pseudomallei* infection or 10 μM CCCP treatment by western blot.

(**b**) Determined of Parkin aggregated on mitochondria in RAW264.7 cells with *B. pseudomallei* infection or CCCP treatment.

(**c**) Quantification of Pink1 mRNA expression in RAW264.7 cells. Cells were treated as in (**b**).

(**d**) Quantification of *Parkin* and *Pink1* mRNA expression in *Parkin* or *Pink1* knock down RAW264.7 cells.

(**e** and **f**) Western blot analysis of Parkin or PINK1 protein level in *Parkin* or *Pink1* knock down RAW264.7 cells, respectively.

(**g**) Confocal microscopy images of HSP60 colocalized with Ub in RAW264.7 cells. Cells were treated as in (**b**), and labelled for HSP60 (HSP60 antibody, red) and Ub (FK2 antibody, green). Scale bar, 5 μm.

(**h**) Quantification of the Ub colocalized with mitochondria in (**e**).

(**i**) qPCR analysis of mtDNA/nDNA levels in *Parkin* and *Pink1* knock down RAW264.7 cells. Cells were treated as in (**b**).

(**j** and **k**) Detection of the mRNA and protein expression levels of Nlrx1, Nix, Bnip3 and Fundc1 in RAW264.7 cells after transfected with the indicated siRNA, respectively.

(**l**) Quantification of mtDNA/nDNA levels of the cells in (**j**) after *B. pseudomallei* infection. The above data were from at least 3 independent experiments and showed means ± SD. One-way ANOVA followed by Tukey post hoc test (**c**, **i**, **l**), two-tailed Student's *t* test (**d**, **j**) and two-way ANOVA followed by Tukey post hoc test (**h**) were used for data analysis. n = 3 in each group (**a**, **d**, **h**, **i**, **j**, **l**). Source data were provided as a Source Data file.

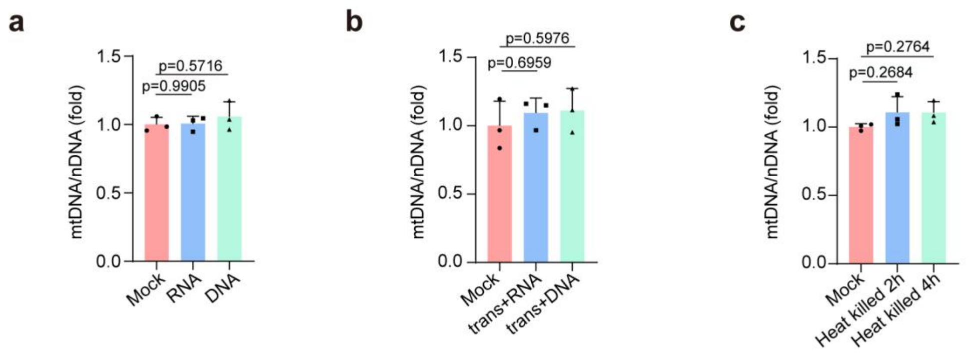

Supplementary Figure 4. Mitophagy is actively modulated by *B. pseudomallei* infection in macrophages

(**a-c**) Quantification of mtDNA/nDNA levels in RAW264.7 cells. Cells were treated with (**a**) RNA or DNA of *B. pseudomallei* or (**b**) RNA or DNA of *B. pseudomallei* by Attractene Transfection Reagents or (**c**) heat killed for the indicated time points. Data were from at least 3 independent experiments and showed means ± SD. trans, transfection. One-way ANOVA followed by Tukey post hoc test was used for data analysis. n = 3 in each group. Source data were provided as a Source Data file.

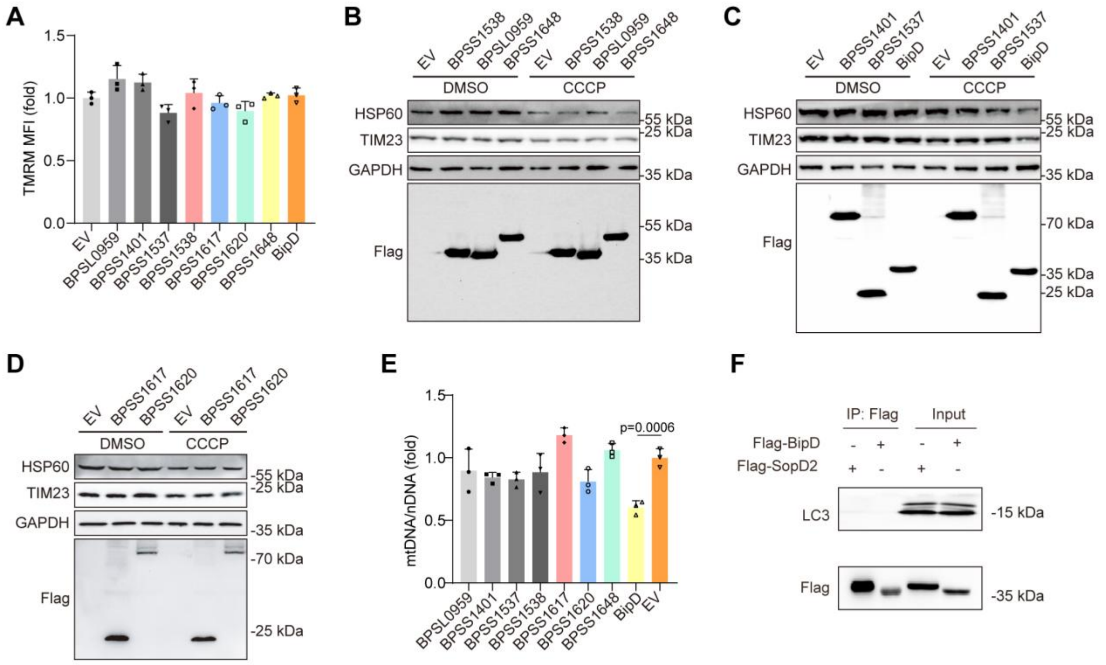

Supplementary Figure 5. Measurement on the possible function of predicted proteins of *B. pseudomallei* involved in the mitophagy induction

(**a**) TMRM staining and flow cytometry analysis the effect of the 8 predicted proteins on Δψm in HEK293T cells. TMRM fluorescence intensity was quantified from 3 independent experiments, and showed means ± SD. Constructed the Flag-tagged plasmids of the 8 predicted proteins of *B. pseudomallei* listed in Supplementary Data 1.

(**b-d**) Determined the effect of the 8 predicted proteins on mitophagy in HEK293T cells in the absence or presence of CCCP (10 μM) by western blot analysis with anti-HSP60, anti-TIM23, anti-GAPDH and anti-Flag.

(**e**) Determined the effect of the 8 predicted proteins on mtDNA/nDNA ratio in HEK293T cells by qPCR analysis. Data were showed means ± SD from 3 different experiments.

(**f**) CoIP analysis of the BipD colocalization with LC3 in HEK293T cells. Cells were transfected with Flag-BipD or Flag-SopD2 plasmid for 24 h, respectively. Results characteristic of three separate trials. One-way ANOVA followed by Tukey post hoc test (**a**, **e**) was used for data analysis. n = 3 in each group (**a**, **e**). Source data were provided as a Source Data file.

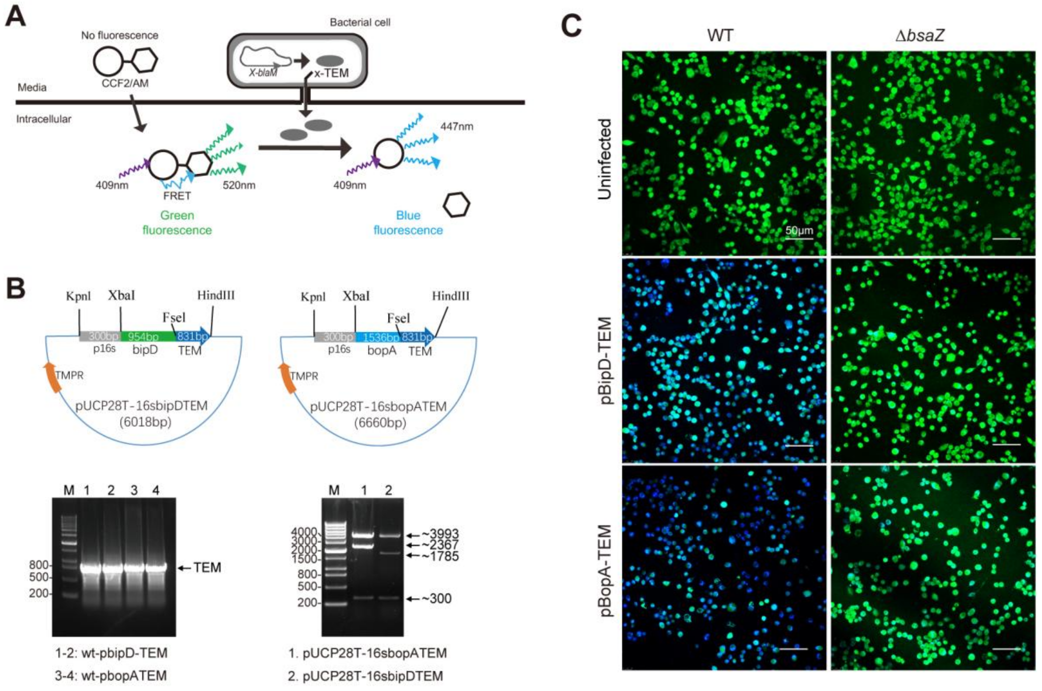

Supplementary Figure 6. Assay of T3SS-dependent secretion of BipD in RAW264.7 cells

(**a**) The schematic diagram of TEM-1 beta-lactamase reporter system.

(**b**) Constructed the plasmids for TEM-1 beta-lactamase reporter system. The gene of bipD was driven by p16s promoter in pUCP28T, and the fusion expression plasmid was detected by PCR and the execution of *Kpn* I, *Xba* I and *Hin*d III three enzyme digestion. BopA was a known protein secreted by the T3SS of *B. pseudomallei*.

(**c**) Representative confocal immunofluorescence microscopy images of RAW264.7 cells infected with the *B. pseudomallei* strains expressing the plasmids in (**b**). Cells were observed at 447 nm (blue) or 520 nm (green) light. Blue fluorescence was observed on infection with *B. pseudomallei* carrying Bla fusions of BopA (positive control), and BipD. Green fluorescence was observed in RAW264.7 cells infected with Δ*bsaZ* strains (lacking T3SS secretion function) or uninfected cells. Scale bar, 50 μm. Results characteristic of three separate trials. Source data were provided as a Source Data file.

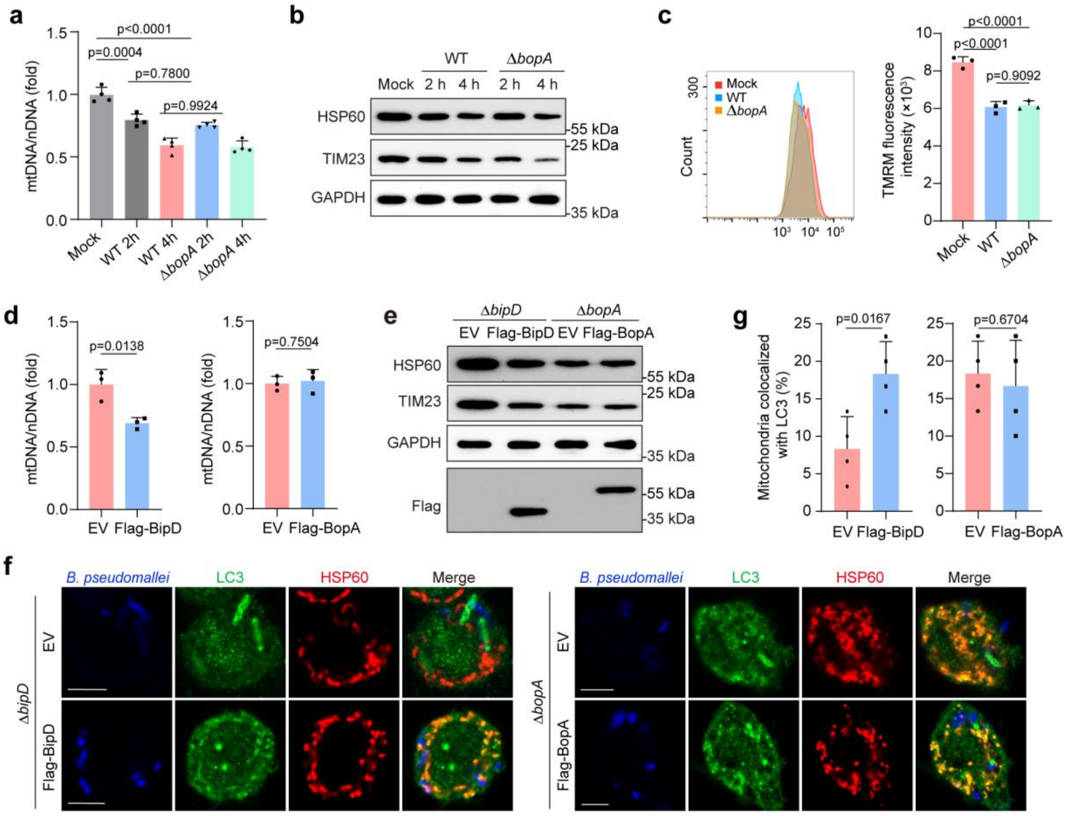

Supplementary Figure 7. BopA is not involved in *B. pseudomallei*-induced mitophagy

(**a**) Quantification of mtDNA/nDNA levels in RAW264.7 cells upon infection of *B. pseudomallei* WT or Δ*bopA* by qPCR analysis. Cells were infected for different time points (at an MOI of 10).
(**b**) Detection of the mitochondrial proteins HSP60 and TIM23 in *B. pseudomallei* WT or Δ*bopA*-infected RAW264.7 cells. Cells were under treatment in (**a**).
(**c**) Measurement of MMP in *B. pseudomallei* WT or Δ*bopA* infected RAW264.7 cells determined by staining of TMRM. Cells were *B. pseudomallei* infected at an MOI of 10.
(**d**) Quantification of mtDNA/nDNA levels in RAW264.7 cells upon infection of Δ*bipD* or Δ*bopA* *B. pseudomallei* by qPCR analysis. After transfection with EV, Flag-BipD or Flag-BopA plasmids for 24 h, cells were infected with *B. pseudomallei* at an MOI of 10 for additional 4 h.
(**e**) Detection of the mitochondrial proteins HSP60 and TIM23 in Δ*bipD* or Δ*bopA* *B. pseudomallei*-infected RAW264.7 cells. Cells were under treatment in (**d**).
(**f** and **g**) Observation and quantification of colocalizing HSP60 and LC3 in *B. pseudomallei* Δ*bipD* or Δ*bopA* infected RAW264.7 cells. Cells were under treatment in (**d**). Scale bar, 5 μm. The above data were from three or more independent experiments and showed means ± SD. One-way ANOVA followed by Tukey post hoc test (**a**, **c**) and two-tailed Student's *t* test (**d**, **g**) were used for data analysis. n = 3 in each group (**a**, **c**, **d**), n = 4 in each group (**g**). Source data were provided as a Source Data file.

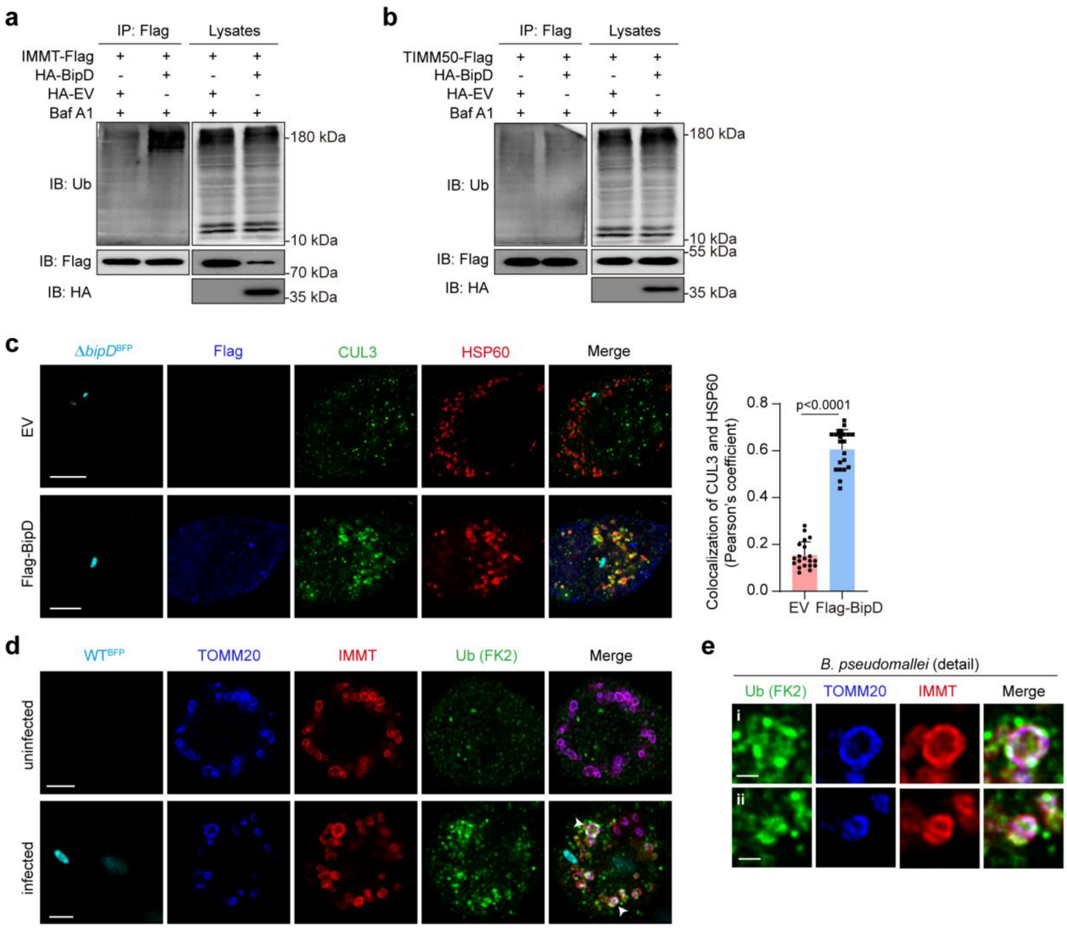

Supplementary Figure 8. Ubiquitination of IMMT in a BipD-dependent manner

(**a** and **b**) Co-IP analysis of ubiquitination of IMMT and TIMM50. HEK293T cells were co-transfected with the HA-EV, HA-BipD and IMMT-Flag or TIMM50-Flag plasmids for 24 h, and then treated with CCCP (10 µM) and Baf A1 (100 nM). Results characteristic of three separate trials.

(**c**) Confocal microscopic imaging of the colocalization of CUL3 (green) with mitochondrial HSP60 (red) in HEK293T cells expressing Flag-BipD (blue) after BFP-labeled *B. pseudomallei* Δ*bipD* (light blue). Scale bars, 5 µm. Pearson's correlation coefficient was used. Data were from 2 independent experiments with 20 cells in each group. Two-tailed Student's *t* test was used for data analysis.

(**d**) RAW264.7 cells were uninfected or infected with BFP-labeled *B. pseudomallei* (light blue) for 4 h, followed by immunolabeled to detect TOMM20 (OMM marker, blue), IMMT (IMM marker, red) and Ub (FK2 antibody, green), and visualized by Airyscan superresolution. White arrowheads indicate examples of discontinuous OMM. Scale bar, 3 µm.

(**e**) High magnifications of mitochondria with discontinuous OMM from *B. pseudomallei* infected RAW264.7 cells. Scale bar, 1 µm. Source data were provided as a Source Data file.

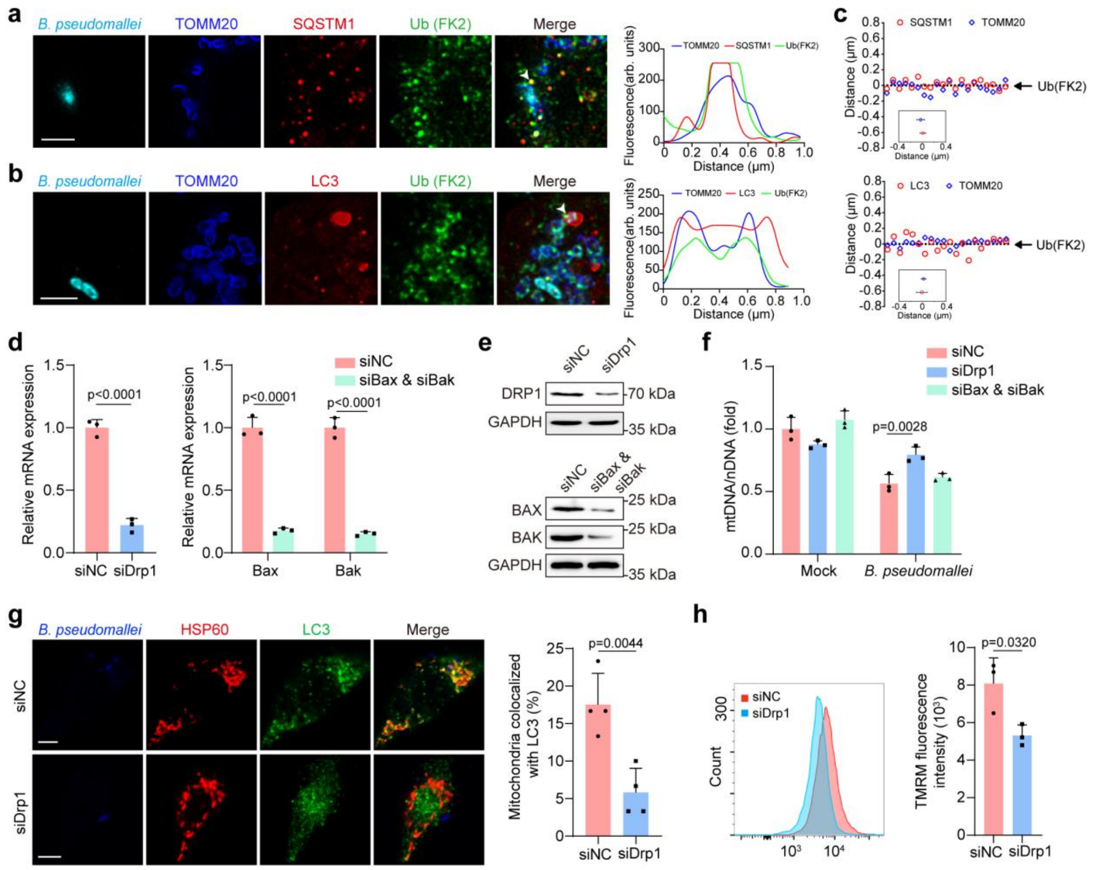

Supplementary Figure 9. Critical roles of SQSTM1 and Drp1 in *B. pseudomallei*-induced mitophagy

(**a** and **b**) RAW264.7 cells were infected with BFP-labeled *B. pseudomallei* for 4 h, followed by labeled with Ub, TOMM20, SQSTM1 or LC3. Fluorescence intensity profiles were shown on the right. Scale bar, 3 μm.

(**c**) Quantification of positions of SQSTM1 and LC3 relative to Ub signal. The insert showed mean ± SD of data shown in the main graph. n=20 per condition was from 2 independent experiments.

(**d** and **e**) Knockdown efficiency of siDrp1, siBax and siBak in RAW264.7 cells was measured, respectively.

(**f**) qPCR detection of mtDNA/nDNA levels in RAW264.7 cells. Cells were treated with siRNA targeting *Drp1*, *Bax* or *Bak*, respectively, and then infected with *B. pseudomallei* for 4 h.

(**g**) Typical confocal images of mitochondria with LC3 after *B. pseudomallei* infection in the presence of siDrp1. Scale bar, 5 μm. Data were from 4 independent experiments with 30 cells in each group.

(**h**) TMRM staining of cells that were treated as in (**g**). The above data were from three or more independent experiments and showed means ± SD. Two-tailed Student's *t* test (**d**, **g**, **h**) and one-way ANOVA (**f**) were used for data analysis. n = 3 in each group (**d**, **f**, **h**), n = 4 in each group (**g**). Source data were provided as a Source Data file.
