# Supplementary material for: Burkholderia pseudomallei BipD modulates host mitophagy to evade killing
Source: Nat Commun. 2024 Jun 4;15:4740. doi: 10.1038/s41467-024-48824-x (PMC11150414; doi:10.1038/s41467-024-48824-x)
Supplement: Supplementary file 3 — Description of Additional Supplementary Files [file 41467_2024_48824_MOESM3_ESM.pdf]

Description of Additional Supplementary Files

File Name: Supplementary Data 1
Description: Predicted LIR motifsa in Type III secrection system proteins of *B. pseudomallei*.

File Name: Supplementary Data 2
Description: Proteins interacting with BipD in HEK293T cells analyzed by IP-MS.

File Name: Supplementary Data 3
Description: Ubiquitin-modified substrates associated with BipD in HEK293T cells by LC-MS/MS analysis.

File Name: Supplementary Data 4
Description: Sequences of primers and plasmids construction used in this study.
