# Supplementary material for: Burkholderia pseudomallei BipD modulates host mitophagy to evade killing
Source: Nat Commun. 2024 Jun 4;15:4740. doi: 10.1038/s41467-024-48824-x (PMC11150414; doi:10.1038/s41467-024-48824-x)
Supplement: Supplementary file 4 — Supplementary Data 1 [file 41467_2024_48824_MOESM4_ESM.docx]

**Supplementary Data 1**

| No. | Gene ID | No. Uniprot | Locus of gene | Function | iLIR(star-end)^b^ | PSSM^c^ |
| --- | --- | --- | --- | --- | --- | --- |
| 1 | BPSL0959 | Q63WC9 | Outside of Annotated T3SS Clusters (1116994-1117959) | Sulfate adenylyltransferase | 76-81;87-92;206-211;212-217;262-267 | 1;9;18;15;2 |
| 2 | BPSS1401 | Q63KF9 | T3SS Cluster 1 (1914705-1916822) | Type III secretion-associated protein | 413-418;63-68;69-74;98-103;114-119;122-127;226-231;286-291;302-307;397-402;498-503;511-516;629-634;678-683;684-689 | 12;4;6;6;2;7;5;7;7;-1;2;5;9;18;10 |
| 3 | BPSS1529 | Q63K37 | T3SS Cluster 3 (2082841-2083773) | Translocator protein BipD; Needle tip protein | 14-19;59-64;145-150;156-161;284-289;305-310 | 18;11;9;5;9;10 |
| 4 | BPSS1537 | Q63K29 | T3SS Cluster 3 (2090401-2091081) | Surface presentation of antigens protein; Inner-membrane export apparatus | 208-213;29-34;71-76;144-149;164-169;219-224 | 20;4;5;5;5;8 |
| 5 | BPSS1538 | Q63K28 | T3SS Cluster 3 (2091071-2092042) | Surface presentation of antigens protein; C-ring component | 56-61;6-11;41-46;48-53;67-72;75-80;103-108 | 21;0;9;11;5;10;13 |
| 6 | BPSS1617 | Q63JU7 | T3SS Cluster 2 (2191756-2192406) | Type III secretion protein | 2-7;28-33;163-168;202-207 | 3;7;5;19 |
| 7 | BPSS1620 | Q63JU4 | T3SS Cluster 2 (2194317-2196389) | Type III secretion protein | 659-664;64-69;70-75;99-104;115-120;123-128;227-232;285-290;293-298;304-309;394-399;492-497;509-514;610-615;665-670 | 19;3;6;8;5;7;5;1;4;7;0;2;7;9;10 |
| 8 | BPSS1648 | Q63JR8 | Outside of Annotated T3SS Clusters (2265019-2266446) | Response regulator | 253-258;350-355;61-66;117-122;133-138;260-265;388-293 | 18;16;6;9;3;-1;11 |

**Supplementary Data 1. Predicted LIR motifs^a^ in Type III secrection system proteins of *B. pseudomallei***

a. A short peptide sequence motif mediated the interaction between autophagy receptors and LC3 is called the LC3-interacting region (LIR) motif.

b. A computational approach for predicting LC3-interaction regions in proteins by the iLIR database.

c. The position-specific scoring matrix of LIR-containing proteins.
