# Supplementary material for: Burkholderia pseudomallei BipD modulates host mitophagy to evade killing
Source: Nat Commun. 2024 Jun 4;15:4740. doi: 10.1038/s41467-024-48824-x (PMC11150414; doi:10.1038/s41467-024-48824-x)
Supplement: Supplementary file 5 — Supplementary Data 2 [file 41467_2024_48824_MOESM5_ESM.docx]

**Supplementary Data 2**

| **Supplementary Data 2. Proteins interacting with BipD in HEK293T cells analyzed by IP-MS** | | | | | |
| --- | --- | --- | --- | --- | --- |
| UniProt Accession | Protein Description | Gene Name | Change | log_2_FC | -log_10_(P_value^*^) |
| P52732 | Kinesin-like protein KIF11 OS=Homo sapiens OX=9606 GN=KIF11 PE=1 SV=2 | KIF11 | up | 5.897718022 | 4.371655669 |
| Q9P2J3 | Kelch-like protein 9 OS=Homo sapiens OX=9606 GN=KLHL9 PE=1 SV=2 | KLHL9 | up | 5.408789146 | 7.846934572 |
| Q9P2N7 | Kelch-like protein 13 OS=Homo sapiens OX=9606 GN=KLHL13 PE=1 SV=3 | KLHL13 | up | 5.14568986 | 5.959474106 |
| P11021 | Endoplasmic reticulum chaperone BiP OS=Homo sapiens OX=9606 GN=HSPA5 PE=1 SV=2 | HSPA5 | up | 3.774760604 | 1.473403066 |
| P14136 | Glial fibrillary acidic protein OS=Homo sapiens OX=9606 GN=GFAP PE=1 SV=1 | GFAP | up | 3.596667715 | 1.607083626 |
| Q9H0A8 | COMM domain-containing protein 4 OS=Homo sapiens OX=9606 GN=COMMD4 PE=1 SV=1 | COMMD4 | up | 3.433041856 | 3.987746125 |
| Q02413 | Desmoglein-1 OS=Homo sapiens OX=9606 GN=DSG1 PE=1 SV=2 | DSG1 | up | 3.232229225 | 1.971240366 |
| P84077 | ADP-ribosylation factor 1 OS=Homo sapiens OX=9606 GN=ARF1 PE=1 SV=2 | ARF1 | up | 2.965659339 | 1.610431147 |
| P14923 | Junction plakoglobin OS=Homo sapiens OX=9606 GN=JUP PE=1 SV=3 | JUP | up | 2.863831805 | 1.850596973 |
| Q4ZG55 | Protein GREB1 OS=Homo sapiens OX=9606 GN=GREB1 PE=2 SV=1 | GREB1 | up | 2.841580554 | 2.437941552 |
| Q969E8 | Pre-rRNA-processing protein TSR2 homolog OS=Homo sapiens OX=9606 GN=TSR2 PE=1 SV=1 | TSR2 | up | 2.799918086 | 7.288597267 |
| P09211 | Glutathione S-transferase P OS=Homo sapiens OX=9606 GN=GSTP1 PE=1 SV=2 | GSTP1 | up | 2.766690483 | 1.611431445 |
| Q96M02 | (E2-independent) E3 ubiquitin-conjugating enzyme FATS OS=Homo sapiens OX=9606 GN=C10orf90 PE=2 SV=2 | C10orf90 | up | 2.584962501 | 3.58531322 |
| Q8IUM7 | Neuronal PAS domain-containing protein 4 OS=Homo sapiens OX=9606 GN=NPAS4 PE=1 SV=1 | NPAS4 | up | 2.574802005 | 6.66461355 |
| P12273 | Prolactin-inducible protein OS=Homo sapiens OX=9606 GN=PIP PE=1 SV=1 | PIP | up | 2.519203899 | 2.498997455 |
| Q9BVG8 | Kinesin-like protein KIFC3 OS=Homo sapiens OX=9606 GN=KIFC3 PE=1 SV=4 | KIFC3 | up | 2.372963274 | 5.589018674 |
| P38646 | Stress-70 protein, mitochondrial OS=Homo sapiens OX=9606 GN=HSPA9 PE=1 SV=2 | HSPA9 | up | 2.351771788 | 1.521342403 |
| P17612 | cAMP-dependent protein kinase catalytic subunit alpha OS=Homo sapiens OX=9606 GN=PRKACA PE=1 SV=2 | PRKACA | up | 2.334962501 | 4.06863244 |
| Q5VZL5 | Zinc finger MYM-type protein 4 OS=Homo sapiens OX=9606 GN=ZMYM4 PE=1 SV=1 | ZMYM4 | up | 2.324802005 | 3.907677483 |
| Q8WZ42 | Titin OS=Homo sapiens OX=9606 GN=TTN PE=1 SV=4 | TTN | up | 2.324802005 | 3.907677483 |
| O95613 | Pericentrin OS=Homo sapiens OX=9606 GN=PCNT PE=1 SV=4 | PCNT | up | 2.316696635 | 1.593809827 |
| P45954 | Short/branched chain specific acyl-CoA dehydrogenase, mitochondrial OS=Homo sapiens OX=9606 GN=ACADSB PE=1 SV=1 | ACADSB | up | 2.29248125 | 1.617335152 |
| Q05823 | 2-5A-dependent ribonuclease OS=Homo sapiens OX=9606 GN=RNASEL PE=1 SV=2 | RNASEL | up | 2.178561379 | 3.820056367 |
| P06702 | Protein S100-A9 OS=Homo sapiens OX=9606 GN=S100A9 PE=1 SV=1 | S100A9 | up | 2.165444524 | 2.714119321 |
| Q7Z3U7 | Protein MON2 homolog OS=Homo sapiens OX=9606 GN=MON2 PE=1 SV=3 | MON2 | up | 2.147178659 | 1.536383125 |
| O95881 | Thioredoxin domain-containing protein 12 OS=Homo sapiens OX=9606 GN=TXNDC12 PE=1 SV=1 | TXNDC12 | up | 2.114857905 | 1.479883318 |
| Q96HU8 | GTP-binding protein Di-Ras2 OS=Homo sapiens OX=9606 GN=DIRAS2 PE=1 SV=1 | DIRAS2 | up | 2.080482024 | 2.610370277 |
| P01040 | Cystatin-A OS=Homo sapiens OX=9606 GN=CSTA PE=1 SV=1 | CSTA | up | 1.872963274 | 2.698069328 |
| P61626 | Lysozyme C OS=Homo sapiens OX=9606 GN=LYZ PE=1 SV=1 | LYZ | up | 1.848079356 | 2.681501207 |
| Q969P5 | F-box only protein 32 OS=Homo sapiens OX=9606 GN=FBXO32 PE=1 SV=1 | FBXO32 | up | 1.848079356 | 1.558529985 |
| P01876 | Immunoglobulin heavy constant alpha 1 OS=Homo sapiens OX=9606 GN=IGHA1 PE=1 SV=2 | IGHA1 | up | 1.834962501 | 1.810951083 |
| P0C0S5 | Histone H2A.Z OS=Homo sapiens OX=9606 GN=H2AZ1 PE=1 SV=2 | H2AZ1 | up | 1.830482024 | 3.15560954 |
| P13639 | Elongation factor 2 OS=Homo sapiens OX=9606 GN=EEF2 PE=1 SV=4 | EEF2 | up | 1.769203899 | 1.72655164 |
| A6NMY6 | Putative annexin A2-like protein OS=Homo sapiens OX=9606 GN=ANXA2P2 PE=5 SV=2 | ANXA2P2 | up | 1.646240625 | 3.359102302 |
| Q5TCS8 | Adenylate kinase 9 OS=Homo sapiens OX=9606 GN=AK9 PE=1 SV=2 | AK9 | up | 1.646240625 | 3.359102302 |
| Q9BXN1 | Asporin OS=Homo sapiens OX=9606 GN=ASPN PE=1 SV=2 | ASPN | up | 1.646240625 | 2.24631253 |
| P62937 | Peptidyl-prolyl cis-trans isomerase A OS=Homo sapiens OX=9606 GN=PPIA PE=1 SV=2 | PPIA | up | 1.580482024 | 1.602437948 |
| Q4G0N8 | Sodium/hydrogen exchanger 10 OS=Homo sapiens OX=9606 GN=SLC9C1 PE=2 SV=2 | SLC9C1 | up | 1.54248125 | 1.498602518 |
| P0DMV8 | Heat shock 70 kDa protein 1A OS=Homo sapiens OX=9606 GN=HSPA1A PE=1 SV=1 | HSPA1A | up | 1.487446453 | 2.874809597 |
| C9JRZ8 | Aldo-keto reductase family 1 member B15 OS=Homo sapiens OX=9606 GN=AKR1B15 PE=1 SV=2 | AKR1B15 | up | 1.476722649 | 2.48164896 |
| P02768 | Albumin OS=Homo sapiens OX=9606 GN=ALB PE=1 SV=2 | ALB | up | 1.455557743 | 1.380337087 |
| Q9NU22 | Midasin OS=Homo sapiens OX=9606 GN=MDN1 PE=1 SV=2 | MDN1 | up | 1.438721876 | 4.196768865 |
| P30048 | Thioredoxin-dependent peroxide reductase, mitochondrial OS=Homo sapiens OX=9606 GN=PRDX3 PE=1 SV=3 | PRDX3 | up | 1.410964047 | 1.332850871 |
| P53804 | E3 ubiquitin-protein ligase TTC3 OS=Homo sapiens OX=9606 GN=TTC3 PE=1 SV=2 | TTC3 | up | 1.396240625 | 1.583559352 |
| P22061 | Protein-L-isoaspartate(D-aspartate) O-methyltransferase OS=Homo sapiens OX=9606 GN=PCMT1 PE=1 SV=4 | PCMT1 | up | 1.372963274 | 1.509074459 |
| P34931 | Heat shock 70 kDa protein 1-like OS=Homo sapiens OX=9606 GN=HSPA1L PE=1 SV=2 | HSPA1L | up | 1.365586561 | 3.012700848 |
| Q2NKX8 | DNA excision repair protein ERCC-6-like OS=Homo sapiens OX=9606 GN=ERCC6L PE=1 SV=1 | ERCC6L | up | 1.25 | 2.610419006 |
| Q13618 | Cullin-3 OS=Homo sapiens OX=9606 GN=CUL3 PE=1 SV=2 | CUL3 | up | 1.25 | 1.397312877 |
| Q6NSI8 | Uncharacterized protein KIAA1841 OS=Homo sapiens OX=9606 GN=KIAA1841 PE=2 SV=2 | KIAA1841 | up | 1.226722649 | 1.333618812 |
| A6NE01 | Protein FAM186A OS=Homo sapiens OX=9606 GN=FAM186A PE=2 SV=3 | FAM186A | up | 1.146240625 | 3.6423 |
| P02649 | Apolipoprotein E OS=Homo sapiens OX=9606 GN=APOE PE=1 SV=1 | APOE | up | 1.146240625 | 3.6423 |
| Q8TES7 | Fas-binding factor 1 OS=Homo sapiens OX=9606 GN=FBF1 PE=1 SV=2 | FBF1 | up | 1.146240625 | 3.6423 |
| Q9BZD4 | Kinetochore protein Nuf2 OS=Homo sapiens OX=9606 GN=NUF2 PE=1 SV=2 | NUF2 | up | 1.146240625 | 3.6423 |
| P05109 | Protein S100-A8 OS=Homo sapiens OX=9606 GN=S100A8 PE=1 SV=1 | S100A8 | up | 1.146240625 | 1.415961825 |
| P53597 | Succinate--CoA ligase [ADP/GDP-forming] subunit alpha, mitochondrial OS=Homo sapiens OX=9606 GN=SUCLG1 PE=1 SV=4 | SUCLG1 | up | 1.146240625 | 1.415961825 |
| P11142 | Heat shock cognate 71 kDa protein OS=Homo sapiens OX=9606 GN=HSPA8 PE=1 SV=1 | HSPA8 | up | 1.117105982 | 1.343078425 |
| P01857 | Immunoglobulin heavy constant gamma 1 OS=Homo sapiens OX=9606 GN=IGHG1 PE=1 SV=1 | IGHG1 | up | 1.04248125 | 1.499773959 |
| P05089 | Arginase-1 OS=Homo sapiens OX=9606 GN=ARG1 PE=1 SV=2 | ARG1 | up | 1.04248125 | 1.499773959 |
| P06733 | Alpha-enolase OS=Homo sapiens OX=9606 GN=ENO1 PE=1 SV=2 | ENO1 | up | 1.04248125 | 1.499773959 |
| P26038 | Moesin OS=Homo sapiens OX=9606 GN=MSN PE=1 SV=3 | MSN | up | 1.04248125 | 1.499773959 |
| Q5VSP4 | Putative lipocalin 1-like protein 1 OS=Homo sapiens OX=9606 GN=LCN1P1 PE=5 SV=1 | LCN1P1 | up | 1.04248125 | 1.499773959 |
| Q8WXH0 | Nesprin-2 OS=Homo sapiens OX=9606 GN=SYNE2 PE=1 SV=3 | SYNE2 | up | 1.04248125 | 1.499773959 |
| P29508 | Serpin B3 OS=Homo sapiens OX=9606 GN=SERPINB3 PE=1 SV=2 | SERPINB3 | up | 1 | 1.302550402 |
| P52292 | Importin subunit alpha-1 OS=Homo sapiens OX=9606 GN=KPNA2 PE=1 SV=1 | KPNA2 | ns | -1.54248125 | 1.289280796 |
| Q9Y6Y0 | Influenza virus NS1A-binding protein OS=Homo sapiens OX=9606 GN=IVNS1ABP PE=1 SV=3 | IVNS1ABP | ns | -2.500703754 | 1.278628051 |
| P10809 | 60 kDa heat shock protein, mitochondrial OS=Homo sapiens OX=9606 GN=HSPD1 PE=1 SV=2 | HSPD1 | ns | -1.29248125 | 1.266851649 |
| P21796 | Voltage-dependent anion-selective channel protein 1 OS=Homo sapiens OX=9606 GN=VDAC1 PE=1 SV=2 | VDAC1 | ns | -2.573080408 | 1.264773357 |
| Q7Z353 | Highly divergent homeobox OS=Homo sapiens OX=9606 GN=HDX PE=1 SV=1 | HDX | ns | -1.701838731 | 1.243362663 |
| Q9UBB4 | Ataxin-10 OS=Homo sapiens OX=9606 GN=ATXN10 PE=1 SV=1 | ATXN10 | ns | -1.701838731 | 1.243362663 |
| P04843 | Dolichyl-diphosphooligosaccharide--protein glycosyltransferase subunit 1 OS=Homo sapiens OX=9606 GN=RPN1 PE=1 SV=1 | RPN1 | ns | -3.256976499 | 1.238078994 |
| P56192 | Methionine--tRNA ligase, cytoplasmic OS=Homo sapiens OX=9606 GN=MARS1 PE=1 SV=2 | MARS1 | ns | -1.744319981 | 1.228353638 |
| Q3KQU3 | MAP7 domain-containing protein 1 OS=Homo sapiens OX=9606 GN=MAP7D1 PE=1 SV=1 | MAP7D1 | ns | -1.5 | 1.2281092 |
| Q15006 | ER membrane protein complex subunit 2 OS=Homo sapiens OX=9606 GN=EMC2 PE=1 SV=1 | EMC2 | ns | -1.79248125 | 1.222674309 |
| Q14498 | RNA-binding protein 39 OS=Homo sapiens OX=9606 GN=RBM39 PE=1 SV=2 | RBM39 | ns | -1.348079356 | 1.213687237 |
| P39656 | Dolichyl-diphosphooligosaccharide--protein glycosyltransferase 48 kDa subunit OS=Homo sapiens OX=9606 GN=DDOST PE=1 SV=4 | DDOST | ns | -1.918106335 | 1.20124179 |
| P31153 | S-adenosylmethionine synthase isoform type-2 OS=Homo sapiens OX=9606 GN=MAT2A PE=1 SV=1 | MAT2A | ns | -1.080482024 | 1.195368429 |
| P46781 | 40S ribosomal protein S9 OS=Homo sapiens OX=9606 GN=RPS9 PE=1 SV=3 | RPS9 | ns | -1.080482024 | 1.195368429 |
| Q96S15 | GATOR complex protein WDR24 OS=Homo sapiens OX=9606 GN=WDR24 PE=1 SV=2 | WDR24 | ns | -1.92510993 | 1.177744993 |
| P19338 | Nucleolin OS=Homo sapiens OX=9606 GN=NCL PE=1 SV=3 | NCL | ns | -3.060925732 | 1.176414116 |
| P55060 | Exportin-2 OS=Homo sapiens OX=9606 GN=CSE1L PE=1 SV=3 | CSE1L | ns | -1.580482024 | 1.170459124 |
| P35232 | Prohibitin OS=Homo sapiens OX=9606 GN=PHB PE=1 SV=1 | PHB | ns | -2.146240625 | 1.15393081 |
| O14980 | Exportin-1 OS=Homo sapiens OX=9606 GN=XPO1 PE=1 SV=1 | XPO1 | ns | -1.848079356 | 1.150517336 |
| Q02878 | 60S ribosomal protein L6 OS=Homo sapiens OX=9606 GN=RPL6 PE=1 SV=3 | RPL6 | ns | -1.744319981 | 1.133969029 |
| Q92841 | Probable ATP-dependent RNA helicase DDX17 OS=Homo sapiens OX=9606 GN=DDX17 PE=1 SV=2 | DDX17 | ns | -1.640560606 | 1.132898811 |
| Q9BUF5 | Tubulin beta-6 chain OS=Homo sapiens OX=9606 GN=TUBB6 PE=1 SV=1 | TUBB6 | ns | -3.202190779 | 1.121441504 |
| O43741 | 5~-AMP-activated protein kinase subunit beta-2 OS=Homo sapiens OX=9606 GN=PRKAB2 PE=1 SV=1 | PRKAB2 | ns | 1.146240625 | 1.121355454 |
| P40925 | Malate dehydrogenase, cytoplasmic OS=Homo sapiens OX=9606 GN=MDH1 PE=1 SV=4 | MDH1 | ns | 1.146240625 | 1.121355454 |
| Q8NF91 | Nesprin-1 OS=Homo sapiens OX=9606 GN=SYNE1 PE=1 SV=4 | SYNE1 | ns | 1.146240625 | 1.121355454 |
| Q86UE4 | Protein LYRIC OS=Homo sapiens OX=9606 GN=MTDH PE=1 SV=2 | MTDH | ns | -1.146240625 | 1.121355454 |
| Q6PJI9 | GATOR complex protein WDR59 OS=Homo sapiens OX=9606 GN=WDR59 PE=1 SV=2 | WDR59 | ns | -2.098079356 | 1.110049003 |
| O75533 | Splicing factor 3B subunit 1 OS=Homo sapiens OX=9606 GN=SF3B1 PE=1 SV=3 | SF3B1 | ns | -2.122294741 | 1.100236265 |
| Q9BVA1 | Tubulin beta-2B chain OS=Homo sapiens OX=9606 GN=TUBB2B PE=1 SV=1 | TUBB2B | ns | -3.931644607 | 1.099465037 |
| P15924 | Desmoplakin OS=Homo sapiens OX=9606 GN=DSP PE=1 SV=3 | DSP | ns | 2.296683322 | 1.087822532 |
| P81605 | Dermcidin OS=Homo sapiens OX=9606 GN=DCD PE=1 SV=2 | DCD | ns | 2.142805285 | 1.071711019 |
| Q07065 | Cytoskeleton-associated protein 4 OS=Homo sapiens OX=9606 GN=CKAP4 PE=1 SV=2 | CKAP4 | ns | -3.015680192 | 1.052293417 |
| P16615 | Sarcoplasmic/endoplasmic reticulum calcium ATPase 2 OS=Homo sapiens OX=9606 GN=ATP2A2 PE=1 SV=1 | ATP2A2 | ns | -3.178776002 | 1.046507963 |
| Q12931 | Heat shock protein 75 kDa, mitochondrial OS=Homo sapiens OX=9606 GN=TRAP1 PE=1 SV=3 | TRAP1 | ns | 1.080482024 | 1.022265753 |
| P60709 | Actin, cytoplasmic 1 OS=Homo sapiens OX=9606 GN=ACTB PE=1 SV=1 | ACTB | ns | -1.889119036 | 0.976479684 |
| P17066 | Heat shock 70 kDa protein 6 OS=Homo sapiens OX=9606 GN=HSPA6 PE=1 SV=2 | HSPA6 | ns | 2.338832691 | 0.961295673 |
| Q08378 | Golgin subfamily A member 3 OS=Homo sapiens OX=9606 GN=GOLGA3 PE=1 SV=2 | GOLGA3 | ns | -1.396240625 | 0.92650081 |
| Q96P63 | Serpin B12 OS=Homo sapiens OX=9606 GN=SERPINB12 PE=1 SV=1 | SERPINB12 | ns | 0.896240625 | 0.908097684 |
| P0CG47 | Polyubiquitin-B OS=Homo sapiens OX=9606 GN=UBB PE=1 SV=1 | UBB | ns | -0.702213746 | 0.824707578 |
| P01591 | Immunoglobulin J chain OS=Homo sapiens OX=9606 GN=JCHAIN PE=1 SV=4 | JCHAIN | ns | 0.646240625 | 0.772421817 |
| P25311 | Zinc-alpha-2-glycoprotein OS=Homo sapiens OX=9606 GN=AZGP1 PE=1 SV=2 | AZGP1 | ns | 0.646240625 | 0.772421817 |
| P16152 | Carbonyl reductase [NADPH] 1 OS=Homo sapiens OX=9606 GN=CBR1 PE=1 SV=3 | CBR1 | ns | 1.532320754 | 0.702263842 |
| P06576 | ATP synthase subunit beta, mitochondrial OS=Homo sapiens OX=9606 GN=ATP5F1B PE=1 SV=3 | ATP5F1B | ns | -1.592017469 | 0.68140746 |
| Q06830 | Peroxiredoxin-1 OS=Homo sapiens OX=9606 GN=PRDX1 PE=1 SV=1 | PRDX1 | ns | 1.795143061 | 0.648421508 |
| P05455 | Lupus La protein OS=Homo sapiens OX=9606 GN=SSB PE=1 SV=2 | SSB | ns | -0.75 | 0.641573804 |
| P08238 | Heat shock protein HSP 90-beta OS=Homo sapiens OX=9606 GN=HSP90AB1 PE=1 SV=4 | HSP90AB1 | ns | -1.743501198 | 0.632769474 |
| P04350 | Tubulin beta-4A chain OS=Homo sapiens OX=9606 GN=TUBB4A PE=1 SV=2 | TUBB4A | ns | -2.540365856 | 0.606896568 |
| O75828 | Carbonyl reductase [NADPH] 3 OS=Homo sapiens OX=9606 GN=CBR3 PE=1 SV=3 | CBR3 | ns | 0.994319981 | 0.569334063 |
| P40227 | T-complex protein 1 subunit zeta OS=Homo sapiens OX=9606 GN=CCT6A PE=1 SV=3 | CCT6A | ns | -0.75 | 0.555587112 |
| Q92945 | Far upstream element-binding protein 2 OS=Homo sapiens OX=9606 GN=KHSRP PE=1 SV=4 | KHSRP | ns | -0.79248125 | 0.537640003 |
| O95816 | BAG family molecular chaperone regulator 2 OS=Homo sapiens OX=9606 GN=BAG2 PE=1 SV=1 | BAG2 | ns | -0.646240625 | 0.510752413 |
| Q14318 | Peptidyl-prolyl cis-trans isomerase FKBP8 OS=Homo sapiens OX=9606 GN=FKBP8 PE=1 SV=2 | FKBP8 | ns | -0.646240625 | 0.510752413 |
| Q6ZN18 | Zinc finger protein AEBP2 OS=Homo sapiens OX=9606 GN=AEBP2 PE=1 SV=2 | AEBP2 | ns | -0.79248125 | 0.451748484 |
| Q86YZ3 | Hornerin OS=Homo sapiens OX=9606 GN=HRNR PE=1 SV=2 | HRNR | ns | -1.429378308 | 0.43006019 |
| Q96DG6 | Carboxymethylenebutenolidase homolog OS=Homo sapiens OX=9606 GN=CMBL PE=1 SV=1 | CMBL | ns | 0.684241399 | 0.397462347 |
| P32119 | Peroxiredoxin-2 OS=Homo sapiens OX=9606 GN=PRDX2 PE=1 SV=5 | PRDX2 | ns | 0.711999227 | 0.393482917 |
| P17844 | Probable ATP-dependent RNA helicase DDX5 OS=Homo sapiens OX=9606 GN=DDX5 PE=1 SV=1 | DDX5 | ns | 0.396240625 | 0.370001163 |
| Q15751 | Probable E3 ubiquitin-protein ligase HERC1 OS=Homo sapiens OX=9606 GN=HERC1 PE=1 SV=2 | HERC1 | ns | 0.396240625 | 0.370001163 |
| P08670 | Vimentin OS=Homo sapiens OX=9606 GN=VIM PE=1 SV=4 | VIM | ns | -0.396240625 | 0.370001163 |
| P46779 | 60S ribosomal protein L28 OS=Homo sapiens OX=9606 GN=RPL28 PE=1 SV=3 | RPL28 | ns | -0.396240625 | 0.370001163 |
| P0C0S8 | Histone H2A type 1 OS=Homo sapiens OX=9606 GN=H2AC11 PE=1 SV=2 | H2AC11 | ns | -0.660964047 | 0.337828279 |
| Q8NHW5 | 60S acidic ribosomal protein P0-like OS=Homo sapiens OX=9606 GN=RPLP0P6 PE=5 SV=1 | RPLP0P6 | ns | 0.353759375 | 0.31985451 |
| P50914 | 60S ribosomal protein L14 OS=Homo sapiens OX=9606 GN=RPL14 PE=1 SV=4 | RPL14 | ns | -0.434241399 | 0.302608676 |
| P05141 | ADP/ATP translocase 2 OS=Homo sapiens OX=9606 GN=SLC25A5 PE=1 SV=7 | SLC25A5 | ns | -0.548161269 | 0.284916485 |
| P07355 | Annexin A2 OS=Homo sapiens OX=9606 GN=ANXA2 PE=1 SV=2 | ANXA2 | ns | -0.5 | 0.275547747 |
| P62826 | GTP-binding nuclear protein Ran OS=Homo sapiens OX=9606 GN=RAN PE=1 SV=3 | RAN | ns | 0.25 | 0.270055376 |
| O94906 | Pre-mRNA-processing factor 6 OS=Homo sapiens OX=9606 GN=PRPF6 PE=1 SV=1 | PRPF6 | ns | -0.25 | 0.270055376 |
| P04792 | Heat shock protein beta-1 OS=Homo sapiens OX=9606 GN=HSPB1 PE=1 SV=2 | HSPB1 | ns | -0.25 | 0.270055376 |
| P62081 | 40S ribosomal protein S7 OS=Homo sapiens OX=9606 GN=RPS7 PE=1 SV=1 | RPS7 | ns | -0.396240625 | 0.23758877 |
| P53618 | Coatomer subunit beta OS=Homo sapiens OX=9606 GN=COPB1 PE=1 SV=3 | COPB1 | ns | 0.353759375 | 0.229270562 |
| P25705 | ATP synthase subunit alpha, mitochondrial OS=Homo sapiens OX=9606 GN=ATP5F1A PE=1 SV=1 | ATP5F1A | ns | -0.62486657 | 0.227982306 |
| Q6UWP8 | Suprabasin OS=Homo sapiens OX=9606 GN=SBSN PE=1 SV=2 | SBSN | ns | 0.29248125 | 0.166760526 |
| P62805 | Histone H4 OS=Homo sapiens OX=9606 GN=H4C1 PE=1 SV=2 | H4C1 | ns | 0.34064252 | 0.152383938 |
| Q08554 | Desmocollin-1 OS=Homo sapiens OX=9606 GN=DSC1 PE=1 SV=2 | DSC1 | ns | 0.25 | 0.136464348 |
| P62906 | 60S ribosomal protein L10a OS=Homo sapiens OX=9606 GN=RPL10A PE=1 SV=2 | RPL10A | ns | -0.392789175 | 0.132829173 |
| P30041 | Peroxiredoxin-6 OS=Homo sapiens OX=9606 GN=PRDX6 PE=1 SV=3 | PRDX6 | ns | -0.226722649 | 0.102213927 |
| Q5D862 | Filaggrin-2 OS=Homo sapiens OX=9606 GN=FLG2 PE=1 SV=1 | FLG2 | ns | -0.25 | 0.093233265 |
| P07900 | Heat shock protein HSP 90-alpha OS=Homo sapiens OX=9606 GN=HSP90AA1 PE=1 SV=5 | HSP90AA1 | ns | -0.20751875 | 0.079357013 |
| Q5UCC4 | ER membrane protein complex subunit 10 OS=Homo sapiens OX=9606 GN=EMC10 PE=1 SV=1 | EMC10 | ns | 0.103759375 | 0.059334604 |
| P62913 | 60S ribosomal protein L11 OS=Homo sapiens OX=9606 GN=RPL11 PE=1 SV=2 | RPL11 | ns | -0.055598105 | 0.022708166 |
| P04406 | Glyceraldehyde-3-phosphate dehydrogenase OS=Homo sapiens OX=9606 GN=GAPDH PE=1 SV=3 | GAPDH | ns | -0.023277351 | 0.02205608 |
| Q9ULH0 | Kinase D-interacting substrate of 220 kDa OS=Homo sapiens OX=9606 GN=KIDINS220 PE=1 SV=3 | KIDINS220 | ns | -0.04248125 | 0.021849921 |
| A6NGG8 | Photoreceptor cilium actin regulator OS=Homo sapiens OX=9606 GN=PCARE PE=1 SV=1 | PCARE | down | 0.896240625 | 1.462635754 |
| O60841 | Eukaryotic translation initiation factor 5B OS=Homo sapiens OX=9606 GN=EIF5B PE=1 SV=4 | EIF5B | down | 0.896240625 | 1.462635754 |
| O76094 | Signal recognition particle subunit SRP72 OS=Homo sapiens OX=9606 GN=SRP72 PE=1 SV=3 | SRP72 | down | 0.896240625 | 1.462635754 |
| P14625 | Endoplasmin OS=Homo sapiens OX=9606 GN=HSP90B1 PE=1 SV=1 | HSP90B1 | down | 0.896240625 | 1.462635754 |
| P43403 | Tyrosine-protein kinase ZAP-70 OS=Homo sapiens OX=9606 GN=ZAP70 PE=1 SV=1 | ZAP70 | down | 0.896240625 | 1.462635754 |
| Q3B7T1 | Erythroid differentiation-related factor 1 OS=Homo sapiens OX=9606 GN=EDRF1 PE=1 SV=1 | EDRF1 | down | 0.896240625 | 1.462635754 |
| Q6WBX8 | Cell cycle checkpoint control protein RAD9B OS=Homo sapiens OX=9606 GN=RAD9B PE=1 SV=2 | RAD9B | down | 0.896240625 | 1.462635754 |
| Q8TE82 | SH3 domain and tetratricopeptide repeat-containing protein 1 OS=Homo sapiens OX=9606 GN=SH3TC1 PE=1 SV=3 | SH3TC1 | down | 0.896240625 | 1.462635754 |
| Q92817 | Envoplakin OS=Homo sapiens OX=9606 GN=EVPL PE=1 SV=3 | EVPL | down | 0.896240625 | 1.462635754 |
| Q96JN2 | Coiled-coil domain-containing protein 136 OS=Homo sapiens OX=9606 GN=CCDC136 PE=1 SV=3 | CCDC136 | down | 0.896240625 | 1.462635754 |
| Q96PY5 | Formin-like protein 2 OS=Homo sapiens OX=9606 GN=FMNL2 PE=1 SV=3 | FMNL2 | down | 0.896240625 | 1.462635754 |
| O43172 | U4/U6 small nuclear ribonucleoprotein Prp4 OS=Homo sapiens OX=9606 GN=PRPF4 PE=1 SV=2 | PRPF4 | down | 0.75 | 1.619640458 |
| O75150 | E3 ubiquitin-protein ligase BRE1B OS=Homo sapiens OX=9606 GN=RNF40 PE=1 SV=5 | RNF40 | down | 0.75 | 1.619640458 |
| P04040 | Catalase OS=Homo sapiens OX=9606 GN=CAT PE=1 SV=3 | CAT | down | 0.75 | 1.619640458 |
| P20930 | Filaggrin OS=Homo sapiens OX=9606 GN=FLG PE=1 SV=3 | FLG | down | 0.75 | 1.619640458 |
| Q12766 | HMG domain-containing protein 3 OS=Homo sapiens OX=9606 GN=HMGXB3 PE=2 SV=2 | HMGXB3 | down | 0.75 | 1.619640458 |
| Q16543 | Hsp90 co-chaperone Cdc37 OS=Homo sapiens OX=9606 GN=CDC37 PE=1 SV=1 | CDC37 | down | 0.75 | 1.619640458 |
| Q16787 | Laminin subunit alpha-3 OS=Homo sapiens OX=9606 GN=LAMA3 PE=1 SV=2 | LAMA3 | down | 0.75 | 1.619640458 |
| Q8IVN3 | Musculoskeletal embryonic nuclear protein 1 OS=Homo sapiens OX=9606 GN=MUSTN1 PE=3 SV=2 | MUSTN1 | down | 0.75 | 1.619640458 |
| Q8IWZ3 | Ankyrin repeat and KH domain-containing protein 1 OS=Homo sapiens OX=9606 GN=ANKHD1 PE=1 SV=1 | ANKHD1 | down | 0.75 | 1.619640458 |
| Q8TF61 | F-box only protein 41 OS=Homo sapiens OX=9606 GN=FBXO41 PE=2 SV=5 | FBXO41 | down | 0.75 | 1.619640458 |
| Q9BYG4 | Partitioning defective 6 homolog gamma OS=Homo sapiens OX=9606 GN=PARD6G PE=1 SV=1 | PARD6G | down | 0.75 | 1.619640458 |
| Q9H254 | Spectrin beta chain, non-erythrocytic 4 OS=Homo sapiens OX=9606 GN=SPTBN4 PE=1 SV=2 | SPTBN4 | down | 0.75 | 1.619640458 |
| Q9NVR5 | Protein kintoun OS=Homo sapiens OX=9606 GN=DNAAF2 PE=1 SV=2 | DNAAF2 | down | 0.75 | 1.619640458 |
| Q9UQE7 | Structural maintenance of chromosomes protein 3 OS=Homo sapiens OX=9606 GN=SMC3 PE=1 SV=2 | SMC3 | down | 0.75 | 1.619640458 |
| Q9Y600 | Cysteine sulfinic acid decarboxylase OS=Homo sapiens OX=9606 GN=CSAD PE=1 SV=2 | CSAD | down | 0.75 | 1.619640458 |
| Q16836 | Hydroxyacyl-coenzyme A dehydrogenase, mitochondrial OS=Homo sapiens OX=9606 GN=HADH PE=1 SV=3 | HADH | down | -0.75 | 1.619640458 |
| Q9NVS9 | Pyridoxine-5~-phosphate oxidase OS=Homo sapiens OX=9606 GN=PNPO PE=1 SV=1 | PNPO | down | -0.75 | 1.619640458 |
| Q10570 | Cleavage and polyadenylation specificity factor subunit 1 OS=Homo sapiens OX=9606 GN=CPSF1 PE=1 SV=2 | CPSF1 | down | -0.896240625 | 1.462635754 |
| Q96HS1 | Serine/threonine-protein phosphatase PGAM5, mitochondrial OS=Homo sapiens OX=9606 GN=PGAM5 PE=1 SV=2 | PGAM5 | down | -0.896240625 | 1.462635754 |
| Q9NY12 | H/ACA ribonucleoprotein complex subunit 1 OS=Homo sapiens OX=9606 GN=GAR1 PE=1 SV=1 | GAR1 | down | -0.896240625 | 1.462635754 |
| Q9NZN8 | CCR4-NOT transcription complex subunit 2 OS=Homo sapiens OX=9606 GN=CNOT2 PE=1 SV=1 | CNOT2 | down | -0.896240625 | 1.462635754 |
| P49411 | Elongation factor Tu, mitochondrial OS=Homo sapiens OX=9606 GN=TUFM PE=1 SV=2 | TUFM | down | -1 | 1.302550402 |
| Q86XN7 | Proline and serine-rich protein 1 OS=Homo sapiens OX=9606 GN=PROSER1 PE=1 SV=2 | PROSER1 | down | -1 | 1.302550402 |
| Q9UKV3 | Apoptotic chromatin condensation inducer in the nucleus OS=Homo sapiens OX=9606 GN=ACIN1 PE=1 SV=2 | ACIN1 | down | -1 | 1.302550402 |
| P10644 | cAMP-dependent protein kinase type I-alpha regulatory subunit OS=Homo sapiens OX=9606 GN=PRKAR1A PE=1 SV=1 | PRKAR1A | down | -1.04248125 | 1.499773959 |
| P62750 | 60S ribosomal protein L23a OS=Homo sapiens OX=9606 GN=RPL23A PE=1 SV=1 | RPL23A | down | -1.04248125 | 1.499773959 |
| Q53GQ0 | Very-long-chain 3-oxoacyl-CoA reductase OS=Homo sapiens OX=9606 GN=HSD17B12 PE=1 SV=2 | HSD17B12 | down | -1.04248125 | 1.499773959 |
| Q96AV8 | Transcription factor E2F7 OS=Homo sapiens OX=9606 GN=E2F7 PE=1 SV=3 | E2F7 | down | -1.04248125 | 1.499773959 |
| Q9Y3Y2 | Chromatin target of PRMT1 protein OS=Homo sapiens OX=9606 GN=CHTOP PE=1 SV=2 | CHTOP | down | -1.04248125 | 1.499773959 |
| O60884 | DnaJ homolog subfamily A member 2 OS=Homo sapiens OX=9606 GN=DNAJA2 PE=1 SV=1 | DNAJA2 | down | -1.146240625 | 1.415961825 |
| P19823 | Inter-alpha-trypsin inhibitor heavy chain H2 OS=Homo sapiens OX=9606 GN=ITIH2 PE=1 SV=2 | ITIH2 | down | -1.146240625 | 1.415961825 |
| P51648 | Aldehyde dehydrogenase family 3 member A2 OS=Homo sapiens OX=9606 GN=ALDH3A2 PE=1 SV=1 | ALDH3A2 | down | -1.146240625 | 1.415961825 |
| P53985 | Monocarboxylate transporter 1 OS=Homo sapiens OX=9606 GN=SLC16A1 PE=1 SV=3 | SLC16A1 | down | -1.146240625 | 1.415961825 |
| O43290 | U4/U6.U5 tri-snRNP-associated protein 1 OS=Homo sapiens OX=9606 GN=SART1 PE=1 SV=1 | SART1 | down | -1.188721876 | 1.619640458 |
| Q5RKV6 | Exosome complex component MTR3 OS=Homo sapiens OX=9606 GN=EXOSC6 PE=1 SV=1 | EXOSC6 | down | -1.188721876 | 1.619640458 |
| P02042 | Hemoglobin subunit delta OS=Homo sapiens OX=9606 GN=HBD PE=1 SV=2 | HBD | down | -1.226722649 | 1.333618812 |
| P54136 | Arginine--tRNA ligase, cytoplasmic OS=Homo sapiens OX=9606 GN=RARS1 PE=1 SV=2 | RARS1 | down | -1.226722649 | 1.333618812 |
| P62191 | 26S proteasome regulatory subunit 4 OS=Homo sapiens OX=9606 GN=PSMC1 PE=1 SV=1 | PSMC1 | down | -1.25 | 1.397312877 |
| Q15046 | Lysine--tRNA ligase OS=Homo sapiens OX=9606 GN=KARS1 PE=1 SV=3 | KARS1 | down | -1.25 | 1.397312877 |
| O95831 | Apoptosis-inducing factor 1, mitochondrial OS=Homo sapiens OX=9606 GN=AIFM1 PE=1 SV=1 | AIFM1 | down | -1.29248125 | 1.577733141 |
| Q5VTE0 | Putative elongation factor 1-alpha-like 3 OS=Homo sapiens OX=9606 GN=EEF1A1P5 PE=5 SV=1 | EEF1A1P5 | down | -1.308576057 | 1.33541875 |
| O60506 | Heterogeneous nuclear ribonucleoprotein Q OS=Homo sapiens OX=9606 GN=SYNCRIP PE=1 SV=2 | SYNCRIP | down | -1.330482024 | 1.351681489 |
| P18031 | Tyrosine-protein phosphatase non-receptor type 1 OS=Homo sapiens OX=9606 GN=PTPN1 PE=1 SV=1 | PTPN1 | down | -1.330482024 | 1.351681489 |
| Q07020 | 60S ribosomal protein L18 OS=Homo sapiens OX=9606 GN=RPL18 PE=1 SV=2 | RPL18 | down | -1.348079356 | 1.341577226 |
| Q8IUR7 | Armadillo repeat-containing protein 8 OS=Homo sapiens OX=9606 GN=ARMC8 PE=1 SV=2 | ARMC8 | down | -1.372963274 | 1.509074459 |
| P04004 | Vitronectin OS=Homo sapiens OX=9606 GN=VTN PE=1 SV=1 | VTN | down | -1.396240625 | 1.583559352 |
| Q13509 | Tubulin beta-3 chain OS=Homo sapiens OX=9606 GN=TUBB3 PE=1 SV=2 | TUBB3 | down | -1.420048865 | 2.901544704 |
| Q5SWX8 | Protein odr-4 homolog OS=Homo sapiens OX=9606 GN=ODR4 PE=1 SV=1 | ODR4 | down | -1.438721876 | 1.444445555 |
| Q9BUJ2 | Heterogeneous nuclear ribonucleoprotein U-like protein 1 OS=Homo sapiens OX=9606 GN=HNRNPUL1 PE=1 SV=2 | HNRNPUL1 | down | -1.438721876 | 1.444445555 |
| O95793 | Double-stranded RNA-binding protein Staufen homolog 1 OS=Homo sapiens OX=9606 GN=STAU1 PE=1 SV=2 | STAU1 | down | -1.476722649 | 1.545336338 |
| P07910 | Heterogeneous nuclear ribonucleoproteins C1/C2 OS=Homo sapiens OX=9606 GN=HNRNPC PE=1 SV=4 | HNRNPC | down | -1.476722649 | 1.545336338 |
| P14678 | Small nuclear ribonucleoprotein-associated proteins B and B~ OS=Homo sapiens OX=9606 GN=SNRPB PE=1 SV=2 | SNRPB | down | -1.54248125 | 1.498602518 |
| Q9NWU2 | Glucose-induced degradation protein 8 homolog OS=Homo sapiens OX=9606 GN=GID8 PE=1 SV=1 | GID8 | down | -1.54248125 | 1.498602518 |
| P26641 | Elongation factor 1-gamma OS=Homo sapiens OX=9606 GN=EEF1G PE=1 SV=3 | EEF1G | down | -1.557204673 | 1.532001156 |
| Q9BQE3 | Tubulin alpha-1C chain OS=Homo sapiens OX=9606 GN=TUBA1C PE=1 SV=1 | TUBA1C | down | -1.612307503 | 3.782193379 |
| Q8N163 | Cell cycle and apoptosis regulator protein 2 OS=Homo sapiens OX=9606 GN=CCAR2 PE=1 SV=2 | CCAR2 | down | -1.622963274 | 1.502820689 |
| P07437 | Tubulin beta chain OS=Homo sapiens OX=9606 GN=TUBB PE=1 SV=2 | TUBB | down | -1.640347607 | 2.923982113 |
| Q99832 | T-complex protein 1 subunit eta OS=Homo sapiens OX=9606 GN=CCT7 PE=1 SV=2 | CCT7 | down | -1.646240625 | 1.568704791 |
| Q86V81 | THO complex subunit 4 OS=Homo sapiens OX=9606 GN=ALYREF PE=1 SV=3 | ALYREF | down | -1.701838731 | 1.531607728 |
| P08865 | 40S ribosomal protein SA OS=Homo sapiens OX=9606 GN=RPSA PE=1 SV=4 | RPSA | down | -1.726722649 | 3.03499281 |
| O15260 | Surfeit locus protein 4 OS=Homo sapiens OX=9606 GN=SURF4 PE=1 SV=3 | SURF4 | down | -1.726722649 | 1.58435121 |
| Q9BPZ2 | Spindlin-2B OS=Homo sapiens OX=9606 GN=SPIN2B PE=1 SV=1 | SPIN2B | down | -1.726722649 | 1.58435121 |
| O95232 | Luc7-like protein 3 OS=Homo sapiens OX=9606 GN=LUC7L3 PE=1 SV=2 | LUC7L3 | down | -1.741446071 | 1.619640458 |
| O75122 | CLIP-associating protein 2 OS=Homo sapiens OX=9606 GN=CLASP2 PE=1 SV=3 | CLASP2 | down | -1.769203899 | 1.408098983 |
| O00571 | ATP-dependent RNA helicase DDX3X OS=Homo sapiens OX=9606 GN=DDX3X PE=1 SV=3 | DDX3X | down | -1.799918086 | 1.45134627 |
| P15880 | 40S ribosomal protein S2 OS=Homo sapiens OX=9606 GN=RPS2 PE=1 SV=2 | RPS2 | down | -1.807204673 | 2.876908076 |
| Q00325 | Phosphate carrier protein, mitochondrial OS=Homo sapiens OX=9606 GN=SLC25A3 PE=1 SV=2 | SLC25A3 | down | -1.807204673 | 1.380824569 |
| Q9P2R3 | Rabankyrin-5 OS=Homo sapiens OX=9606 GN=ANKFY1 PE=1 SV=2 | ANKFY1 | down | -1.848079356 | 1.558529985 |
| Q9UHX1 | Poly(U)-binding-splicing factor PUF60 OS=Homo sapiens OX=9606 GN=PUF60 PE=1 SV=1 | PUF60 | down | -1.848079356 | 1.558529985 |
| P23381 | Tryptophan--tRNA ligase, cytoplasmic OS=Homo sapiens OX=9606 GN=WARS1 PE=1 SV=2 | WARS1 | down | -1.872963274 | 1.394449673 |
| Q9H7D7 | WD repeat-containing protein 26 OS=Homo sapiens OX=9606 GN=WDR26 PE=1 SV=3 | WDR26 | down | -1.872963274 | 1.394449673 |
| Q9Y383 | Putative RNA-binding protein Luc7-like 2 OS=Homo sapiens OX=9606 GN=LUC7L2 PE=1 SV=2 | LUC7L2 | down | -1.910964047 | 1.474281727 |
| O43242 | 26S proteasome non-ATPase regulatory subunit 3 OS=Homo sapiens OX=9606 GN=PSMD3 PE=1 SV=2 | PSMD3 | down | -1.938721876 | 1.40766718 |
| O60825 | 6-phosphofructo-2-kinase/fructose-2,6-bisphosphatase 2 OS=Homo sapiens OX=9606 GN=PFKFB2 PE=1 SV=2 | PFKFB2 | down | -1.951838731 | 1.532844585 |
| P17980 | 26S proteasome regulatory subunit 6A OS=Homo sapiens OX=9606 GN=PSMC3 PE=1 SV=3 | PSMC3 | down | -1.994319981 | 1.515272725 |
| P78371 | T-complex protein 1 subunit beta OS=Homo sapiens OX=9606 GN=CCT2 PE=1 SV=4 | CCT2 | down | -1.994319981 | 1.36199621 |
| P84098 | 60S ribosomal protein L19 OS=Homo sapiens OX=9606 GN=RPL19 PE=1 SV=1 | RPL19 | down | -2.019203899 | 1.397410887 |
| Q15029 | 116 kDa U5 small nuclear ribonucleoprotein component OS=Homo sapiens OX=9606 GN=EFTUD2 PE=1 SV=1 | EFTUD2 | down | -2.019203899 | 1.563932783 |
| Q14974 | Importin subunit beta-1 OS=Homo sapiens OX=9606 GN=KPNB1 PE=1 SV=2 | KPNB1 | down | -2.057204673 | 1.376714189 |
| Q13435 | Splicing factor 3B subunit 2 OS=Homo sapiens OX=9606 GN=SF3B2 PE=1 SV=2 | SF3B2 | down | -2.074802005 | 1.568743606 |
| O43175 | D-3-phosphoglycerate dehydrogenase OS=Homo sapiens OX=9606 GN=PHGDH PE=1 SV=4 | PHGDH | down | -2.098079356 | 1.414980166 |
| O43795 | Unconventional myosin-Ib OS=Homo sapiens OX=9606 GN=MYO1B PE=1 SV=3 | MYO1B | down | -2.105516192 | 1.619640458 |
| Q96S59 | Ran-binding protein 9 OS=Homo sapiens OX=9606 GN=RANBP9 PE=1 SV=1 | RANBP9 | down | -2.12694866 | 1.445491981 |
| Q96EE3 | Nucleoporin SEH1 OS=Homo sapiens OX=9606 GN=SEH1L PE=1 SV=3 | SEH1L | down | -2.157339155 | 1.350736339 |
| P42167 | Lamina-associated polypeptide 2, isoforms beta/gamma OS=Homo sapiens OX=9606 GN=TMPO PE=1 SV=2 | TMPO | down | -2.160964047 | 1.552980676 |
| P26373 | 60S ribosomal protein L13 OS=Homo sapiens OX=9606 GN=RPL13 PE=1 SV=4 | RPL13 | down | -2.202482216 | 1.83354125 |
| Q9P035 | Very-long-chain (3R)-3-hydroxyacyl-CoA dehydratase 3 OS=Homo sapiens OX=9606 GN=HACD3 PE=1 SV=2 | HACD3 | down | -2.203445298 | 1.326914834 |
| Q9Y265 | RuvB-like 1 OS=Homo sapiens OX=9606 GN=RUVBL1 PE=1 SV=1 | RUVBL1 | down | -2.203445298 | 1.548462346 |
| P35613 | Basigin OS=Homo sapiens OX=9606 GN=BSG PE=1 SV=2 | BSG | down | -2.226722649 | 2.974233714 |
| P06748 | Nucleophosmin OS=Homo sapiens OX=9606 GN=NPM1 PE=1 SV=2 | NPM1 | down | -2.226722649 | 1.423721759 |
| Q12906 | Interleukin enhancer-binding factor 3 OS=Homo sapiens OX=9606 GN=ILF3 PE=1 SV=3 | ILF3 | down | -2.269203899 | 1.427278626 |
| P18124 | 60S ribosomal protein L7 OS=Homo sapiens OX=9606 GN=RPL7 PE=1 SV=1 | RPL7 | down | -2.288704527 | 1.909163868 |
| P49368 | T-complex protein 1 subunit gamma OS=Homo sapiens OX=9606 GN=CCT3 PE=1 SV=4 | CCT3 | down | -2.330482024 | 1.611669863 |
| P07477 | Trypsin-1 OS=Homo sapiens OX=9606 GN=PRSS1 PE=1 SV=1 | PRSS1 | down | -2.340554454 | 2.812009018 |
| P50991 | T-complex protein 1 subunit delta OS=Homo sapiens OX=9606 GN=CCT4 PE=1 SV=4 | CCT4 | down | -2.341580554 | 1.570751166 |
| Q9NP73 | Putative bifunctional UDP-N-acetylglucosamine transferase and deubiquitinase ALG13 OS=Homo sapiens OX=9606 GN=ALG13 PE=1 SV=2 | ALG13 | down | -2.349685923 | 1.49054653 |
| Q9Y230 | RuvB-like 2 OS=Homo sapiens OX=9606 GN=RUVBL2 PE=1 SV=3 | RUVBL2 | down | -2.372963274 | 1.510767047 |
| Q6VN20 | Ran-binding protein 10 OS=Homo sapiens OX=9606 GN=RANBP10 PE=1 SV=1 | RANBP10 | down | -2.387686696 | 1.491832054 |
| Q13310 | Polyadenylate-binding protein 4 OS=Homo sapiens OX=9606 GN=PABPC4 PE=1 SV=1 | PABPC4 | down | -2.532320754 | 1.587971658 |
| Q08211 | ATP-dependent RNA helicase A OS=Homo sapiens OX=9606 GN=DHX9 PE=1 SV=4 | DHX9 | down | -2.548073204 | 1.462534125 |
| P62917 | 60S ribosomal protein L8 OS=Homo sapiens OX=9606 GN=RPL8 PE=1 SV=2 | RPL8 | down | -2.574802005 | 2.765285253 |
| Q9UM54 | Unconventional myosin-VI OS=Homo sapiens OX=9606 GN=MYO6 PE=1 SV=4 | MYO6 | down | -2.574802005 | 1.535564521 |
| Q99623 | Prohibitin-2 OS=Homo sapiens OX=9606 GN=PHB2 PE=1 SV=2 | PHB2 | down | -2.604463129 | 1.374748869 |
| P83731 | 60S ribosomal protein L24 OS=Homo sapiens OX=9606 GN=RPL24 PE=1 SV=1 | RPL24 | down | -2.726722649 | 5.478797604 |
| P05388 | 60S acidic ribosomal protein P0 OS=Homo sapiens OX=9606 GN=RPLP0 PE=1 SV=1 | RPLP0 | down | -2.730088214 | 1.491609956 |
| P17987 | T-complex protein 1 subunit alpha OS=Homo sapiens OX=9606 GN=TCP1 PE=1 SV=1 | TCP1 | down | -2.769203899 | 2.365545114 |
| P47914 | 60S ribosomal protein L29 OS=Homo sapiens OX=9606 GN=RPL29 PE=1 SV=2 | RPL29 | down | -2.79248125 | 2.042590004 |
| Q8WXF1 | Paraspeckle component 1 OS=Homo sapiens OX=9606 GN=PSPC1 PE=1 SV=1 | PSPC1 | down | -2.799918086 | 1.616994049 |
| P31689 | DnaJ homolog subfamily A member 1 OS=Homo sapiens OX=9606 GN=DNAJA1 PE=1 SV=2 | DNAJA1 | down | -2.890560606 | 4.099744931 |
| P50990 | T-complex protein 1 subunit theta OS=Homo sapiens OX=9606 GN=CCT8 PE=1 SV=4 | CCT8 | down | -2.890560606 | 3.862569516 |
| P01023 | Alpha-2-macroglobulin OS=Homo sapiens OX=9606 GN=A2M PE=1 SV=3 | A2M | down | -2.928561379 | 1.56264401 |
| P07814 | Bifunctional glutamate/proline--tRNA ligase OS=Homo sapiens OX=9606 GN=EPRS1 PE=1 SV=5 | EPRS1 | down | -2.932829758 | 1.377193825 |
| Q5VT06 | Centrosome-associated protein 350 OS=Homo sapiens OX=9606 GN=CEP350 PE=1 SV=1 | CEP350 | down | -2.953445298 | 1.600210232 |
| P59190 | Ras-related protein Rab-15 OS=Homo sapiens OX=9606 GN=RAB15 PE=1 SV=1 | RAB15 | down | -2.956438458 | 3.182191325 |
| Q9H082 | Ras-related protein Rab-33B OS=Homo sapiens OX=9606 GN=RAB33B PE=1 SV=1 | RAB33B | down | -2.956438458 | 3.182191325 |
| Q15233 | Non-POU domain-containing octamer-binding protein OS=Homo sapiens OX=9606 GN=NONO PE=1 SV=4 | NONO | down | -2.973704441 | 1.616718485 |
| O00159 | Unconventional myosin-Ic OS=Homo sapiens OX=9606 GN=MYO1C PE=1 SV=4 | MYO1C | down | -3.005591953 | 1.60629748 |
| Q00839 | Heterogeneous nuclear ribonucleoprotein U OS=Homo sapiens OX=9606 GN=HNRNPU PE=1 SV=6 | HNRNPU | down | -3.007839149 | 1.414797122 |
| P68371 | Tubulin beta-4B chain OS=Homo sapiens OX=9606 GN=TUBB4B PE=1 SV=1 | TUBB4B | down | -3.014791277 | 1.385142036 |
| O15294 | UDP-N-acetylglucosamine--peptide N-acetylglucosaminyltransferase 110 kDa subunit OS=Homo sapiens OX=9606 GN=OGT PE=1 SV=3 | OGT | down | -3.016690483 | 1.403690446 |
| Q9Y277 | Voltage-dependent anion-selective channel protein 3 OS=Homo sapiens OX=9606 GN=VDAC3 PE=1 SV=1 | VDAC3 | down | -3.04248125 | 3.255056373 |
| O43143 | Pre-mRNA-splicing factor ATP-dependent RNA helicase DHX15 OS=Homo sapiens OX=9606 GN=DHX15 PE=1 SV=2 | DHX15 | down | -3.056301859 | 1.6122221 |
| P45880 | Voltage-dependent anion-selective channel protein 2 OS=Homo sapiens OX=9606 GN=VDAC2 PE=1 SV=2 | VDAC2 | down | -3.068303202 | 1.573765957 |
| O14654 | Insulin receptor substrate 4 OS=Homo sapiens OX=9606 GN=IRS4 PE=1 SV=1 | IRS4 | down | -3.08272922 | 1.505541016 |
| Q6N021 | Methylcytosine dioxygenase TET2 OS=Homo sapiens OX=9606 GN=TET2 PE=1 SV=3 | TET2 | down | -3.141810776 | 1.615695122 |
| P55795 | Heterogeneous nuclear ribonucleoprotein H2 OS=Homo sapiens OX=9606 GN=HNRNPH2 PE=1 SV=1 | HNRNPH2 | down | -3.182086429 | 1.578791572 |
| P48643 | T-complex protein 1 subunit epsilon OS=Homo sapiens OX=9606 GN=CCT5 PE=1 SV=1 | CCT5 | down | -3.185998215 | 4.946898765 |
| O75688 | Protein phosphatase 1B OS=Homo sapiens OX=9606 GN=PPM1B PE=1 SV=1 | PPM1B | down | -3.211372513 | 2.42878922 |
| P62753 | 40S ribosomal protein S6 OS=Homo sapiens OX=9606 GN=RPS6 PE=1 SV=1 | RPS6 | down | -3.231203126 | 2.807774627 |
| Q9NZI8 | Insulin-like growth factor 2 mRNA-binding protein 1 OS=Homo sapiens OX=9606 GN=IGF2BP1 PE=1 SV=2 | IGF2BP1 | down | -3.288704527 | 1.615992666 |
| Q9UH99 | SUN domain-containing protein 2 OS=Homo sapiens OX=9606 GN=SUN2 PE=1 SV=3 | SUN2 | down | -3.307204673 | 1.537681251 |
| P67809 | Y-box-binding protein 1 OS=Homo sapiens OX=9606 GN=YBX1 PE=1 SV=3 | YBX1 | down | -3.328787391 | 4.959557229 |
| P61026 | Ras-related protein Rab-10 OS=Homo sapiens OX=9606 GN=RAB10 PE=1 SV=1 | RAB10 | down | -3.339525427 | 1.616409797 |
| P62424 | 60S ribosomal protein L7a OS=Homo sapiens OX=9606 GN=RPL7A PE=1 SV=2 | RPL7A | down | -3.348481669 | 3.101487338 |
| P46777 | 60S ribosomal protein L5 OS=Homo sapiens OX=9606 GN=RPL5 PE=1 SV=3 | RPL5 | down | -3.355516192 | 4.838976857 |
| Q15393 | Splicing factor 3B subunit 3 OS=Homo sapiens OX=9606 GN=SF3B3 PE=1 SV=4 | SF3B3 | down | -3.371268641 | 1.616138817 |
| O43318 | Mitogen-activated protein kinase kinase kinase 7 OS=Homo sapiens OX=9606 GN=MAP3K7 PE=1 SV=1 | MAP3K7 | down | -3.409269414 | 5.271131254 |
| P62820 | Ras-related protein Rab-1A OS=Homo sapiens OX=9606 GN=RAB1A PE=1 SV=3 | RAB1A | down | -3.41942991 | 1.500103882 |
| Q01804 | OTU domain-containing protein 4 OS=Homo sapiens OX=9606 GN=OTUD4 PE=1 SV=4 | OTUD4 | down | -3.520869832 | 1.608599401 |
| P61006 | Ras-related protein Rab-8A OS=Homo sapiens OX=9606 GN=RAB8A PE=1 SV=1 | RAB8A | down | -3.632388141 | 1.594949635 |
| Q9NRW1 | Ras-related protein Rab-6B OS=Homo sapiens OX=9606 GN=RAB6B PE=1 SV=1 | RAB6B | down | -3.642167173 | 2.642749905 |
| P61978 | Heterogeneous nuclear ribonucleoprotein K OS=Homo sapiens OX=9606 GN=HNRNPK PE=1 SV=1 | HNRNPK | down | -3.654825739 | 4.688623032 |
| Q5W0B1 | ORC ubiquitin ligase 1 OS=Homo sapiens OX=9606 GN=OBI1 PE=1 SV=1 | OBI1 | down | -3.661667801 | 1.584235134 |
| P23458 | Tyrosine-protein kinase JAK1 OS=Homo sapiens OX=9606 GN=JAK1 PE=1 SV=2 | JAK1 | down | -3.698705493 | 1.571270269 |
| P62701 | 40S ribosomal protein S4, X isoform OS=Homo sapiens OX=9606 GN=RPS4X PE=1 SV=2 | RPS4X | down | -3.735766052 | 6.739126454 |
| Q9BQA1 | Methylosome protein 50 OS=Homo sapiens OX=9606 GN=WDR77 PE=1 SV=1 | WDR77 | down | -3.783476197 | 2.836820679 |
| Q9NXC5 | GATOR complex protein MIOS OS=Homo sapiens OX=9606 GN=MIOS PE=1 SV=2 | MIOS | down | -3.785845803 | 1.508807222 |
| P52272 | Heterogeneous nuclear ribonucleoprotein M OS=Homo sapiens OX=9606 GN=HNRNPM PE=1 SV=3 | HNRNPM | down | -3.954383332 | 4.069692806 |
| P62241 | 40S ribosomal protein S8 OS=Homo sapiens OX=9606 GN=RPS8 PE=1 SV=2 | RPS8 | down | -3.995023735 | 5.14032063 |
| Q6IQ22 | Ras-related protein Rab-12 OS=Homo sapiens OX=9606 GN=RAB12 PE=1 SV=3 | RAB12 | down | -4.009043403 | 3.004945812 |
| P36578 | 60S ribosomal protein L4 OS=Homo sapiens OX=9606 GN=RPL4 PE=1 SV=5 | RPL4 | down | -4.014723422 | 3.084327043 |
| P11940 | Polyadenylate-binding protein 1 OS=Homo sapiens OX=9606 GN=PABPC1 PE=1 SV=2 | PABPC1 | down | -4.059451868 | 5.264833283 |
| A0A075B6S2 | Immunoglobulin kappa variable 2D-29 OS=Homo sapiens OX=9606 GN=IGKV2D-29 PE=3 SV=1 | IGKV2D-29 | down | -4.115497738 | 1.907356698 |
| P61247 | 40S ribosomal protein S3a OS=Homo sapiens OX=9606 GN=RPS3A PE=1 SV=2 | RPS3A | down | -4.139848007 | 6.472150003 |
| P20340 | Ras-related protein Rab-6A OS=Homo sapiens OX=9606 GN=RAB6A PE=1 SV=3 | RAB6A | down | -4.148785226 | 1.605484653 |
| P39023 | 60S ribosomal protein L3 OS=Homo sapiens OX=9606 GN=RPL3 PE=1 SV=2 | RPL3 | down | -4.162247366 | 5.274900197 |
| P31943 | Heterogeneous nuclear ribonucleoprotein H OS=Homo sapiens OX=9606 GN=HNRNPH1 PE=1 SV=4 | HNRNPH1 | down | -4.223289826 | 3.933619074 |
| Q9Y2H1 | Serine/threonine-protein kinase 38-like OS=Homo sapiens OX=9606 GN=STK38L PE=1 SV=3 | STK38L | down | -4.226370256 | 3.227377444 |
| O15397 | Importin-8 OS=Homo sapiens OX=9606 GN=IPO8 PE=1 SV=2 | IPO8 | down | -4.434757253 | 1.588249864 |
| P23396 | 40S ribosomal protein S3 OS=Homo sapiens OX=9606 GN=RPS3 PE=1 SV=2 | RPS3 | down | -4.536622281 | 6.030461812 |
| Q15208 | Serine/threonine-protein kinase 38 OS=Homo sapiens OX=9606 GN=STK38 PE=1 SV=1 | STK38 | down | -4.593432951 | 3.315940445 |
| P23588 | Eukaryotic translation initiation factor 4B OS=Homo sapiens OX=9606 GN=EIF4B PE=1 SV=2 | EIF4B | down | -4.600653101 | 4.28871861 |
| Q16875 | 6-phosphofructo-2-kinase/fructose-2,6-bisphosphatase 3 OS=Homo sapiens OX=9606 GN=PFKFB3 PE=1 SV=1 | PFKFB3 | down | -4.770387763 | 4.196011301 |
| Q15750 | TGF-beta-activated kinase 1 and MAP3K7-binding protein 1 OS=Homo sapiens OX=9606 GN=TAB1 PE=1 SV=1 | TAB1 | down | -4.928561379 | 10.35231894 |
| P54105 | Methylosome subunit pICln OS=Homo sapiens OX=9606 GN=CLNS1A PE=1 SV=1 | CLNS1A | down | -5.191703392 | 2.482718105 |
| Q8WWY3 | U4/U6 small nuclear ribonucleoprotein Prp31 OS=Homo sapiens OX=9606 GN=PRPF31 PE=1 SV=2 | PRPF31 | down | -5.257752977 | 6.903604421 |
| Q9Y657 | Spindlin-1 OS=Homo sapiens OX=9606 GN=SPIN1 PE=1 SV=3 | SPIN1 | down | -5.47104263 | 7.706060091 |
| Q9BUA3 | Spindlin interactor and repressor of chromatin-binding protein OS=Homo sapiens OX=9606 GN=SPINDOC PE=1 SV=3 | SPINDOC | down | -5.699767035 | 4.723130543 |
| Q9Y2W1 | Thyroid hormone receptor-associated protein 3 OS=Homo sapiens OX=9606 GN=THRAP3 PE=1 SV=2 | THRAP3 | down | -5.811646012 | 2.747056017 |
| Q9NYF8 | Bcl-2-associated transcription factor 1 OS=Homo sapiens OX=9606 GN=BCLAF1 PE=1 SV=2 | BCLAF1 | down | -5.850623957 | 5.421252166 |
| Q9BRS2 | Serine/threonine-protein kinase RIO1 OS=Homo sapiens OX=9606 GN=RIOK1 PE=1 SV=2 | RIOK1 | down | -6.0882929 | 8.920899646 |
| P98175 | RNA-binding protein 10 OS=Homo sapiens OX=9606 GN=RBM10 PE=1 SV=3 | RBM10 | down | -6.364099258 | 3.948196214 |
| O14744 | Protein arginine N-methyltransferase 5 OS=Homo sapiens OX=9606 GN=PRMT5 PE=1 SV=4 | PRMT5 | down | -7.905175835 | 5.815765425 |

*: Two-tailed Student’s *t* test were used for data analysis.

up: up-regulated.

down: down-regulated.

ns: no significance.
