# Supplementary material for: Burkholderia pseudomallei BipD modulates host mitophagy to evade killing
Source: Nat Commun. 2024 Jun 4;15:4740. doi: 10.1038/s41467-024-48824-x (PMC11150414; doi:10.1038/s41467-024-48824-x)
Supplement: Supplementary file 6 — Supplementary Data 3 [file 41467_2024_48824_MOESM6_ESM.docx]

**Supplementary Data 3**

| **Supplementary Data 3. Ubiquitin-modified substrates associated with BipD in HEK293T cells by LC-MS/MS analysis** | | | | |
| --- | --- | --- | --- | --- |
| UniProt Accession | Protein Description | Gene Name | log_2_FC | -log_10_(P-value^*^) |
| Q8N490 | Probable hydrolase PNKD OS=Homo sapiens OX=9606 GN=PNKD PE=1 SV=2 | PNKD | 3.437405312 | 2.239024717 |
| P30041 | Peroxiredoxin-6 OS=Homo sapiens OX=9606 GN=PRDX6 PE=1 SV=3 | PRDX6 | 2.807354922 | 3.921683734 |
| Q16891 | MICOS complex subunit MIC60 OS=Homo sapiens OX=9606 GN=IMMT PE=1 SV=1 | IMMT | 2.765534746 | 4.476234926 |
| P82930 | 28S ribosomal protein S34, mitochondrial OS=Homo sapiens OX=9606 GN=MRPS34 PE=1 SV=2 | MRPS34 | 2.760049207 | 3.391422051 |
| P29372 | DNA-3-methyladenine glycosylase OS=Homo sapiens OX=9606 GN=MPG PE=1 SV=3 | MPG | 2.748742762 | 2.781170546 |
| O75746 | Calcium-binding mitochondrial carrier protein Aralar1 OS=Homo sapiens OX=9606 GN=SLC25A12 PE=1 SV=2 | SLC25A12 | 2.491853096 | 2.157025688 |
| O75146 | Huntingtin-interacting protein 1-related protein OS=Homo sapiens OX=9606 GN=HIP1R PE=1 SV=2 | HIP1R | 2.409390936 | 1.965228487 |
| P47897 | Glutamine--tRNA ligase OS=Homo sapiens OX=9606 GN=QARS1 PE=1 SV=1 | QARS1 | 2.226770862 | 1.849577808 |
| Q7Z4H7 | HAUS augmin-like complex subunit 6 OS=Homo sapiens OX=9606 GN=HAUS6 PE=1 SV=2 | HAUS6 | 2.129283017 | 1.611994331 |
| Q5T9A4 | ATPase family AAA domain-containing protein 3B OS=Homo sapiens OX=9606 GN=ATAD3B PE=1 SV=1 | ATAD3B | 1.990084531 | 1.690246209 |
| Q08J23 | RNA cytosine C(5)-methyltransferase NSUN2 OS=Homo sapiens OX=9606 GN=NSUN2 PE=1 SV=2 | NSUN2 | 1.964666927 | 1.538297981 |
| O00116 | Alkyldihydroxyacetonephosphate synthase, peroxisomal OS=Homo sapiens OX=9606 GN=AGPS PE=1 SV=1 | AGPS | 1.932885804 | 1.897691005 |
| Q96QK1 | Vacuolar protein sorting-associated protein 35 OS=Homo sapiens OX=9606 GN=VPS35 PE=1 SV=2 | VPS35 | 1.809634662 | 1.307021421 |
| P36776 | Lon protease homolog, mitochondrial OS=Homo sapiens OX=9606 GN=LONP1 PE=1 SV=2 | LONP1 | 1.711494907 | 1.537408185 |
| P51398 | 28S ribosomal protein S29, mitochondrial OS=Homo sapiens OX=9606 GN=DAP3 PE=1 SV=1 | DAP3 | 1.652076697 | 1.305019861 |
| O95573 | Long-chain-fatty-acid--CoA ligase 3 OS=Homo sapiens OX=9606 GN=ACSL3 PE=1 SV=3 | ACSL3 | 1.64385619 | 1.464850793 |
| Q9Y2R9 | 28S ribosomal protein S7, mitochondrial OS=Homo sapiens OX=9606 GN=MRPS7 PE=1 SV=2 | MRPS7 | 1.631612594 | 1.463900735 |
| Q3ZCQ8 | Mitochondrial import inner membrane translocase subunit TIM50 OS=Homo sapiens OX=9606 GN=TIMM50 PE=1 SV=2 | TIMM50 | 1.584962501 | 1.552566146 |
| P49588 | Alanine--tRNA ligase, cytoplasmic OS=Homo sapiens OX=9606 GN=AARS1 PE=1 SV=2 | AARS1 | 1.514573173 | 1.997414527 |
| P34932 | Heat shock 70 kDa protein 4 OS=Homo sapiens OX=9606 GN=HSPA4 PE=1 SV=4 | HSPA4 | 1.072669068 | 1.582308784 |
| Q13501 | Sequestosome-1 OS=Homo sapiens OX=9606 GN=SQSTM1 PE=1 SV=1 | SQSTM1 | 0.857259828 | 1.350144983 |
| P62699 | Protein yippee-like 5 OS=Homo sapiens OX=9606 GN=YPEL5 PE=1 SV=1 | YPEL5 | 1.795180208 | 1.44661039 |
| P0DMV8 | Heat shock 70 kDa protein 1A OS=Homo sapiens OX=9606 GN=HSPA1A PE=1 SV=1 | HSPA1A | 4.042348015 | 1.60949052 |

*: Two-tailed Student’s *t* test were used for data analysis.
