# Supplementary material for: Burkholderia pseudomallei BipD modulates host mitophagy to evade killing
Source: Nat Commun. 2024 Jun 4;15:4740. doi: 10.1038/s41467-024-48824-x (PMC11150414; doi:10.1038/s41467-024-48824-x)
Supplement: Supplementary file 7 — Supplementary Data 4 [file 41467_2024_48824_MOESM7_ESM.docx]

**Supplementary Data 4**

| **Supplementary Data 4. Sequences of primers and plasmids construction used in this study** | | |
| --- | --- | --- |
| Use | Name | Sequence (5'-3') |
| mtDNA/nDNA qPCR | M-mtAtp6-F | TTCCACTATGAGCTGGAGCCGTAAT |
|  | M-mtAtp6-R | CGGACTGCTAATGCCATTGGTTGA |
|  | M-Rlp13a-F | GGGCAGGTTCTGGTATTGGAT |
|  | M-Rlp13a-R | GGCTCGGAAATGGTAGGGG |
|  |  |  |
| Gene expression qPCR | Pink1-qpF | TTCTTCCGCCAGTCGGTAG |
|  | Pink1-qpR | CTGCTTCTCCTCGATCAGCC |
|  | Parkin-qpF | GAGGTCGATTCTGACACCAGC |
|  | Parkin-qpR | CCGGCAAAAATCACACGCAG |
|  | Fundc1-qpF | AGCGATGACGAATCATACGAAG |
|  | Fundc1-qpR | CCACCCATTACAATCTGAGTAGC |
|  | Nlrx1-qpF | TAGGGCCTTTATCCGTTACCA |
|  | Nlrx1-qpF | TAAACCACTCGGTGAGGTTCC |
|  | Bnip3l-qpF | CTGGAGCACGTTCCTTCCTC |
|  | Bnip3l-qpR | ACAGTGCGAACTGCCTCTTG |
|  | Drp1-qpF | GGTGGTGGGATTGGAGA |
|  | Drp1-qpR | CCTTTCTGTGGACTTGCTG |
|  | Bax-qpF | GCCTCGCTCACCATCTG |
|  | Bax-qpR | CCCACCCCTCCCAATAA |
|  | Bak-qpF | TGGCATCTGGACAAGGAC |
|  | Bak-qpR | CCTGCTGGTGGAGGTAAAA |
|  | Atg5-qpF | CTGCGGTTCACTCTGGTT |
|  | Atg5-qpR | TCGGTCGGGTTCTGTCT |
|  | Atg7-qpF | GCAGTTTCCAGTCCGTTG |
|  | Atg7-qpR | GGTTCTTCTCCCAGCCA |
|  | FIP200-qpF | TGCTCTCTCCTGATATGCC |
|  | FIP200-qpR | CCTAAGTTCTTTGCCACGTT |
|  | Bnip3-qpF | CTGGGTAGAACTGCACTTCAG |
|  | Bnip3-qpR | GGAGCTACTTCGTCCAGATTCAT |
|  | Gapdh-qpF | GGATGCTGCCCTTACCC |
|  | Gapdh-qpR | GTTCACACCGACCTTCACC |
|  | Cul3-qpF | TCAGTCAGCCACACCAAA |
|  | Cul3-qpR | AACACCAACCTCAGATCCA |
|  | Klhl9-qpF | GGTGTCTCTGGGTAACGGC |
|  | Klhl9-qpR | AACTGTGCGTGTTGCTGGTAA |
|  | Klhl13-qpF | AGAATTGGTTGCTGCAATACTCC |
|  | Klhl13-qpR | AAGGCACAGTTTCAAGTGCTG |
|  | Immt-qpF | GAAATGCCTACCATCCCA |
|  | Immt-qpR | GGTTTCGTCAATCATCGC |
|  |  |  |
| KLHL9 deletion mutants construction | pcDNA4His-F | GGAATTCTGCAGATATCCAGCACAGT |
|  | pcDNA4His-R | AGGTACCTTATCGTCATCGTCGTACAGA |
|  | pcK9BTB-F | TGACGATAAGGTACCTATGAAAGTGTCCCTTGGTAACGG |
|  | pcK9BTB-R | ATCTGCAGAATTCCAGAGACTCCTGATATAAGAAATACTTTACAGAAATC |
|  | pcK9BACK-F | TGACGATAAGGTACCTTTGGATAACTGTGTTGAGGTTGGACGA |
|  | pcK9BACK-R | ATATCTGCAGAATTCCTGGCATCATTTGGTAATTGCTAGCTTCCA |
|  | pcK9Kelch-F | TGACGATAAGGTACCTTATATGCAGCCAGTGATGCAGTCAGATAG |
|  | pcK9Kelch-R | ATATCTGCAGAATTCCCTAAGAATGATCTGAAGGTGCTGAAAGAGGT |
|  |  |  |
| KLHL13 deletion mutants construction | pcK13BTB-F | TGACGATAAGGTACCTATGATGAGAGTTCAAACCTTAAGAGAAAAATG |
|  | pcK13BTB-R | ATATCTGCAGAATTCCGACCCCAGATATGAGAAACACTTTACAGAA |
|  | pcK13BACK-F | TGACGATAAGGTACCTACTTTAGACAACTGTGTTGAAGTTGGACG |
|  | pcK13BACK-R | ATATCTGCAGAATTCCTTGCACGTAATTAATGAGCTCCTGTGG |
|  | pcK13Kelch-F | TGACGATAAGGTACCTACGGTGGATTTCATGAGAACTGACAAT |
|  | pcK13Kelch-R | ATATCTGCAGAATTCCTTAAGGTGCAGAAAGAGGGGACTCTC |
|  |  |  |
| IMMT K122R construction | pcDNA4-IMMT-K122R-F | TGTATCAGAAGTAATGAAAGAATCTAGACAGCCTGCCTCAC |
|  | pcDNA4-IMMT-K122R-R | GTGAGGCAGGCTGTCTAGATTCTTTCATTACTTCTGATACA |
| IMMT K211R construction | pcDNA4-IMMT-K211R-F | GCTCGCCTTGCACAACAGGAAAGACAAGAACAAGTTAAAATT |
|  | pcDNA4-IMMT-K211R-R | AATTTTAACTTGTTCTTGTCTTTCCTGTTGTGCAAGGCGAGC |
| IMMT K216R construction | pcDNA4-IMMT-K216R-F | CAGGAAAAACAAGAACAAGTTAGAATTGAGTCTCTAGCCAAGAGC |
|  | pcDNA4-IMMT-K216R-R | GCTCTTGGCTAGAGACTCAATTCTAACTTGTTCTTGTTTTTCCTG |
| IMMT K370R construction | pcDNA4-IMMT-K370R-F | CCAAGCTCGGGATGACTTTAGACGAGAGCTGG |
|  | pcDNA4-IMMT-K370R-R | CCAGCTCTCGTCTAAAGTCATCCCGAGCTTGG |
| IMMT K467R construction | pcDNA4-IMMT-K467R-F | GGCTGAACAGGACAGAAGGATAGAAGAAGTCAGAG |
|  | pcDNA4-IMMT-K467R-R | CTCTGACTTCTTCTATCCTTCTGTCCTGTTCAGCC |
| IMMT K516R construction | pcDNA4-IMMT-K516R-F | GAATTTGAGCAGAACCTGTCTGAGAGACTCTCTGAACAA |
|  | pcDNA4-IMMT-K516R-R | TTGTTCAGAGAGTCTCTCAGACAGGTTCTGCTCAAATTC |
|  |  |  |
| cloning bipD into pK18mobsacB | BipD-up-F | CTATGACATGATTACGAATTCCGAGACGTCGTTGAGCAACTGATCG |
|  | BipD-up-R | GTGAACCACGACGAGGCCGCGCCCACGC |
|  | BipD-down-F | GCGGCCTCATCGTGGTTCACCTTCGTTCTATCG |
|  | BipD-down-R | ACGACGGCCAGTGCCAAGCTTTCAAGCAGTTCCACGCGG |
|  |  |  |
| shRNA | shCul3(M) | GATAGAAAGTGGCCACATATT |
|  | shKlhl9(M) | CCCTGTTCTAACCTAATATAA |
|  | shKlhl13(M) | CGTCGATACAGTGTTTAGATT |
|  | shCUL3(H) | CGTAAGAATAACAGTGGTCTT |
|  | shKLHL9(H) | CCGTTTCTATTCAAATGGAAA |
|  | shKLHL13(H) | GAATGGACCTATGTTGCCAAA |
|  | shPINK1(H) | CGGCTGGAGGAGTATCTGATA |
|  | shPRKN(H) | CGTGATTTGCTTAGACTGTTT |
|  | shScramble | CCTAAGGTTAAGTCGCCCTCG |
|  | shPink1(M） | CCTGGCTGACTATCCTGATAT |
|  | shParkin(M） | CGTTTCATTATCTGCAACTTT |
|  |  |  |
| siRNA | siNlrx1-1999 | GACUACUACAAUGACGAUGUUTT |
|  | siBnip3-404 | AGAAGUUGAAAGUAUCCUGAATT |
|  | siNix-221 | GCAAUGGCAAUGAGAAUGGAATT |
|  | siFundc1-481 | GAUAAAGAAGCGAGCAAAUAATT |
|  | siDrp1 | CGAGAUUGUGAGGUUAUUGAATT |
|  | siBax | UAUGGAGCUGCAGAGGAUGTT |
|  | siBak | UGCCUACGAACUCUUCACCTT |
|  | siFip200 | GCUGAAUUUCAGUGCUUAGAATT |
|  | siAtg5 | GCAUUAUCCAAUUGGUUUATT |
|  | siAtg7 | CAGUGGAUCUAAAUCUCAAACUGAUTT |
|  |  |  |
| sgRNA | sgCul3 | TATGTCTCTAATCATCACCATGG |
|  | sgImmt | TTTCTTCTAGGTTGACTGC |
|  |  |  |
| cloning bipD into pUCP28T | bipD-F | TTTCTAGAATGAACATGCATGTCGACATGGG |
|  | bipDgfp-R | GTGGATCCCGGGCCCGCGGTACCGTCGACTGCAGGATCTGCAGATAGCTCTTGGCG |
|  |  |  |
