# Supplementary material for: Burkholderia pseudomallei BipD modulates host mitophagy to evade killing
Source: Nat Commun. 2024 Jun 4;15:4740. doi: 10.1038/s41467-024-48824-x (PMC11150414; doi:10.1038/s41467-024-48824-x)
Supplement: Supplementary file 8 — Reporting Summary [file 41467_2024_48824_MOESM8_ESM.pdf]

nature portfolio

Corresponding author(s): Qian Li

Last updated by author(s): Apr 22, 2024

Reporting Summary

Nature Portfolio wishes to improve the reproducibility of the work that we publish. This form provides structure for consistency and transparency in reporting. For further information on Nature Portfolio policies, see our Editorial Policies and the Editorial Policy Checklist.

Statistics

For all statistical analyses, confirm that the following items are present in the figure legend, table legend, main text, or Methods section.

n/a	Confirmed	
☐	☒	The exact sample size (*n*) for each experimental group/condition, given as a discrete number and unit of measurement
☐	☒	A statement on whether measurements were taken from distinct samples or whether the same sample was measured repeatedly
☐	☒	The statistical test(s) used AND whether they are one- or two-sided<br>*Only common tests should be described solely by name; describe more complex techniques in the Methods section.*
☒	☐	A description of all covariates tested
☐	☒	A description of any assumptions or corrections, such as tests of normality and adjustment for multiple comparisons
☐	☒	A full description of the statistical parameters including central tendency (e.g. means) or other basic estimates (e.g. regression coefficient) AND variation (e.g. standard deviation) or associated estimates of uncertainty (e.g. confidence intervals)
☐	☒	For null hypothesis testing, the test statistic (e.g. *F*, *t*, *r*) with confidence intervals, effect sizes, degrees of freedom and *P* value noted<br>*Give P values as exact values whenever suitable.*
☒	☐	For Bayesian analysis, information on the choice of priors and Markov chain Monte Carlo settings
☒	☐	For hierarchical and complex designs, identification of the appropriate level for tests and full reporting of outcomes
☐	☒	Estimates of effect sizes (e.g. Cohen's *d*, Pearson's *r*), indicating how they were calculated

Our web collection on statistics for biologists contains articles on many of the points above.

Software and code

Policy information about availability of computer code

Data collection	No software was used for data collection.
Data analysis	The images of blot and gels were analyzed using Image Lab™ Software (version 6.0.0, build 25, Bio-Rad USA).The Confocal images were analyzed by Leica Application Suite Las X software (v2.0.1.14392). The data of flow cytometry were analyzed using FlowJo software v10 and GraphPad Prism (v8.0). The raw files of mass spectrometry were processed with Mascot Daemon (version 2.3.02, Matrix Science). Statistical analysis was performed using GraphPad Prism (v8.0) and ImageJ 1.53e software.

For manuscripts utilizing custom algorithms or software that are central to the research but not yet described in published literature, software must be made available to editors and reviewers. We strongly encourage code deposition in a community repository (e.g. GitHub). See the Nature Portfolio guidelines for submitting code & software for further information.

Data

Policy information about availability of data

All manuscripts must include a data availability statement. This statement should provide the following information, where applicable:

- Accession codes, unique identifiers, or web links for publicly available datasets
- A description of any restrictions on data availability
- For clinical datasets or third party data, please ensure that the statement adheres to our policy

All relevant data are included in the manuscript and its supplementary information files. Soure data are provided in Source Data file. The proteomics data used in this study are available in the ProteomeXchange database at PXD051631. Source data are provided with this paper.

Research involving human participants, their data, or biological material

Policy information about studies with human participants or human data. See also policy information about sex, gender (identity/presentation), and sexual orientation and race, ethnicity and racism.

Reporting on sex and gender	N/A. This study only used animal experiments without using human participants or human data.
Reporting on race, ethnicity, or other socially relevant groupings	N/A. This study only used animal experiments without using human participants or human data.
Population characteristics	N/A. This study only used animal experiments without using human participants or human data.
Recruitment	N/A. Human research participants and clinical data were not involved in our study.
Ethics oversight	N/A. Human research participants and clinical data were not involved in our study.

Note that full information on the approval of the study protocol must also be provided in the manuscript.

Field-specific reporting

Please select the one below that is the best fit for your research. If you are not sure, read the appropriate sections before making your selection.

☒ Life sciences ☐ Behavioural & social sciences ☐ Ecological, evolutionary & environmental sciences

For a reference copy of the document with all sections, see nature.com/documents/nr-reporting-summary-flat.pdf

Life sciences study design

All studies must disclose on these points even when the disclosure is negative.

Sample size	The sample sizes were chosen based on the similar reported in previous publications. For Western blot, qPCR, flow cytometry and mass spectrometry assays, n = 3. For intracellular bactrial load assays in cells, livers or spleens from animals, n = 5 or n = 6. For survival ratio assays of animal experiments, n = 12.
Data exclusions	No data were excluded from the analyses
Replication	All assays data in the study were performance at least three biological replicates, and the details were provided in the figure legends. The validity of the experiments was successfully reproduced in each case.
Randomization	All samples were randomly divided into each group.
Blinding	Blinding was done during data collection and analysis.

Reporting for specific materials, systems and methods

We require information from authors about some types of materials, experimental systems and methods used in many studies. Here, indicate whether each material, system or method listed is relevant to your study. If you are not sure if a list item applies to your research, read the appropriate section before selecting a response.

Materials & experimental systems

n/a	Involved in the study
☐	☒ Antibodies
☐	☒ Eukaryotic cell lines
☒	☐ Palaeontology and archaeology
☐	☒ Animals and other organisms
☒	☐ Clinical data
☒	☐ Dual use research of concern
☒	☐ Plants

Methods

n/a	Involved in the study
☒	☐ ChIP-seq
☐	☒ Flow cytometry
☒	☐ MRI-based neuroimaging

Antibodies

Antibodies used

For western blot, coimmunoprecipitation and immunofluorescence assay, HSP60 Mouse Monoclonal Antibody (Beyotime, Cat#AF0186), HSP60 Rabbit Monoclonal Antibody (Beyotime, Cat#AF1771), GAPDH (D16H11) XP® Rabbit mAb (CST, Cat#5174, RRID: AB_10622025), Laboratories™ Purified Mouse Anti-TIM23 (BD Biosciences, Cat#611222, RRID: AB_398754), LC3A/LC3B Polyclonal Antibody (Thermo Fisher Scientific, Cat#PA1-16931, RRID: AB_2137583), Donkey anti-Mouse IgG (H+L) Highly Cross-Adsorbed Secondary Antibody, Alexa Fluor™ 594 (Thermo Fisher Scientific, Cat# A-21203, RRID: AB_141633), Goat anti-Rabbit IgG (H+L) Cross-Adsorbed Secondary Antibody, Alexa Fluor™ 568 Thermo (Fisher Scientific, Cat#A-11011, RRID: AB_143157), Flag Tag Rabbit mAb (Zenbio, Cat#R24091), Rabbit anti GST-Tag mAb (Abclonal, Cat#AE077), Anti-KLHL9 antibody (Abcam, Cat#ab230542), KLHL13 Polyclonal Antibody (Thermo Fisher Scientific, Cat#PA5-31658, RRID: AB_2549131), Xpress Monoclonal Antibody (Thermo Fisher Scientific, Cat#R910-25, RRID: AB_2556552), Anti-Ubiquitinylated proteins Antibody (clone FK2, Millipore, Cat#04-263, RRID: AB_612093), Anti-Cullin 3 antibody (Abcam, Cat#ab75851, RRID: AB_1523423), Ubiquitin antibody (P4D1) (Santa Cruz Biotechnology, Cat#sc-8017, RRID: AB_628423), Anti-DnaK Antibody (Abcam, Cat#ab69617, RRID: AB_1209209), Anti-Mitofilin antibody (Abcam, Cat#ab137057), Myc Tag Mouse Monoclonal Antibody (Beyotime, Cat#AF0033, RRID: AB_2939055), HA tag antibody (Thermo Fisher Scientific, Cat#26183, RRID: AB_10978021), LAMP1 Monoclonal Antibody (LY1C6) (Thermo Fisher Scientific, Cat# MA1-164, RRID: AB_2536869), anti-KIF5B (Abcam, Cat#ab167429), anti-DYNLL1 (Abcam, Cat#ab51603), anti-PINK1 (CST, Cat#6946), anti-Parkin (Thermo Fisher Scientific, Cat#PA513399), anti-NLRX1 (ABclonal, Cat#A4976), anti-BNIP3L (Zenbio, Cat#381891), anti-BNIP3 (Zenbio, Cat#R23308), anti-FUNDC1 (Zenbio, Cat#251933), anti-ATG5 (CST, Cat#12994T), anti-ATG7 (CST, Cat#8558T), anti-FIP200 (Abclonal, Cat#A14685), anti-BAX (Zenbio, Cat#R22708), anti-BAK (Abclonal, Cat#A0204), anti-DRP1 (Abclonal, Cat#A2586), anti-SQSTM1 (Invitrogen, Cat#MA5-27800, RRID:AB_2735371), anti-TOMM20 (Abcam, Cat#ab186735, RRID:AB_2889972), anti-rabbit IgG, HRP-linked Antibody (CST, Cat#7074, RRID: AB_2099233), Anti-mouse IgG, HRP-linked Antibody (CST, Cat#7076, RRID: AB_330924).

Validation

Antibodies targeted the following proteins were used in this study: HSP60 Mouse Monoclonal Antibody (Beyotime, AF0186, https://www.beyotime.com/product/AF0186.htm), HSP60 Rabbit Monoclonal Antibody (Beyotime, AF1771, https://www.beyotime.com/product/AF1771.htm), GAPDH (CST, 5174, https://www.cellsignal.cn/products/primary-antibodies/gapdh-d16h11-xp-174-rabbit-mab/5174), TIM23 (clone 32, BD Biosciences, 611222, https://www.bdbiosciences.com/zh-cn/products/reagents/microscopy-imaging-reagents/immunofluorescence-reagents/purified-mouse-anti-tim23.611222), LC3A/LC3B (Thermo Fisher Scientific, PA1-16931, https://www.thermofisher.cn/cn/zh/antibody/product/LC3A-LC3B-Antibody-Polyclonal/PA1-16931), Flag (Zenbio, R24091, http://www.zen-bio.cn/prod_view.aspx?IsActiveTarget=True&TypeId=239&Id=639631&FId=t3:239:3), SQSTM1 (Invitrogen, MA5-27800, https://www.thermofisher.cn/cn/zh/antibody/product/SQSTM1-Antibody-clone-GT1478-Monoclonal/MA5-27800), TOMM20 (Abcam, ab186735, https://www.abcam.cn/products/primary-antibodies/tomm20-antibody-epr15581-54-mitochondrial-marker-ab186735.html), GST (Abclonal, AE077, https://abclonal.com.cn/catalog/AE077), KLHL9 (Abcam, ab230542, https://www.abcam.cn/products/primary-antibodies/klhl9-antibody-ab230542.html), KLHL13 (Thermo Fisher Scientific, PA5-31658, https://www.thermofisher.cn/cn/zh/antibody/product/KLHL13-Antibody-Polyclonal/PA5-31658), Xpress (Thermo Fisher Scientific, R910-25, https://www.thermofisher.cn/cn/zh/antibody/product/Xpress-Antibody-Monoclonal/R910-25), Ubiquitinylated proteins (clone FK2, Millipore, 04-263, https://www.sigmaaldrich.cn/CN/zh/product/mm/04263), Cullin 3 (Abcam, ab75851, https://www.abcam.cn/products/primary-antibodies/cullin-3cul-3-antibody-epr3196y-ab75851.html), Ubiquitin antibody (clone P4D1, Santa Cruz Biotechnology, sc-8017, https://www.scbt.com/zh/p/ubiquitin-antibody-p4d1), DnaK (Abcam, ab69617, https://www.abcam.cn/products/primary-antibodies/dnak-antibody-8e22-ab69617.html), Mitofilin (Abcam, ab137057, https://www.abcam.cn/products/primary-antibodies/mitofilin-antibody-epr8749-ab137057.html), Myc (Beyotime, AF0033, https://www.beyotime.com/product/AF0033.htm), HA (Thermo Fisher Scientific, 26183, https://www.thermofisher.cn/cn/zh/antibody/product/HA-Tag-Antibody-clone-2-2-2-14-Monoclonal/26183), LAMP1 (Thermo Fisher Scientific, MA1-164, https://www.thermofisher.cn/cn/zh/antibody/product/LAMP1-Antibody-clone-LY1C6-Monoclonal/MA1-164). KIF5B (Abcam, ab167429, https://www.abcam.cn/products/primary-antibodies/kif5bkif5c-antibody-epr10276b-ab167429.html), DYNLL1 (Abcam, ab51603, https://www.abcam.cn/products/primary-antibodies/dynll1pin-antibody-ep1660y-ab51603.html), ATG5 (CST, 12994T, https://www.cellsignal.cn/products/primary-antibodies/atg5-d5f5u-rabbit-mab/12994), ATG7 (CST, 8558T, https://www.cellsignal.cn/products/primary-antibodies/atg7-d12b11-rabbit-mab/8558), FIP200 (Abclonal, A14685, https://abclonal.com.cn/catalog/A14685), PINK1 (CST, 6946, https://www.cellsignal.cn/products/primary-antibodies/pink1-d8g3-rabbit-mab/6946), Parkin (Thermo Fisher Scientific, PA513399, https://www.thermofisher.cn/cn/zh/antibody/product/Parkin-Antibody-Polyclonal/PA5-13399), NLRX1 (ABclonal, A4976, https://abclonal.com.cn/catalog/A4976), BNIP3L (Zenbio, 381891, http://www.zen-bio.cn/prod_view.aspx?IsActiveTarget=True&TypeId=246&Id=685210&FId=t3:246:3), BNIP3 (Zenbio, R23308, http://www.zen-bio.cn/prod_view.aspx?IsActiveTarget=True&TypeId=245&Id=686134&FId=t3:245:3), FUNDC1 (Zenbio, 251933, http://www.zen-bio.cn/prod_view.aspx?IsActiveTarget=True&TypeId=246&Id=674974&FId=t3:246:3), BAX (Zenbio, R22708, http://www.zen-bio.cn/prod_view.aspx?IsActiveTarget=True&TypeId=245&Id=655887&FId=t3:245:3), BAK (Abclonal, A0204, https://abclonal.com.cn/catalog/A0204), DRP1 (Abclonal, A2586,https://abclonal.com.cn/catalog/A2586), Donkey anti-Mouse IgG (H+L) Highly Cross-Adsorbed Secondary Antibody, Alexa Fluor™ 594 (A-21203, https://www.thermofisher.cn/cn/zh/antibody/product/Donkey-anti-Mouse-IgG-H-L-Highly-Cross-Adsorbed-Secondary-Antibody-Polyclonal/A-21203) and Goat anti-Rabbit IgG (H+L) Cross-Adsorbed Secondary Antibody, Alexa Fluor™ 568 (A-11011, https://www.thermofisher.cn/cn/zh/antibody/product/Goat-anti-Rabbit-IgG-H-L-Cross-Adsorbed-Secondary-Antibody-Polyclonal/A-11011) were purchased from Thermo Fisher Scientific . Anti-rabbit IgG, HRP-linked Antibody (7074, https://www.cellsignal.cn/products/secondary-antibodies/anti-rabbit-igg-hrp-linked-antibody/7074) and anti-mouse IgG, HRP-linked

Antibody (7076, https://www.cellsignal.cn/products/secondary-antibodies/anti-mouse-igg-hrp-linked-antibody/7076) were purchased from CST.

Eukaryotic cell lines

Policy information about cell lines and Sex and Gender in Research

Cell line source(s)	HEK293T cells (ATCC Cat: tings-161813; RRID: CVCL_0063) and RAW264.7 cells (ATCC Cat: tings-12733; RRID: CVCL_0493) were purchased from ATCC. RAW264.7-EGFP-LC3-PURO cells were purchased from HANBIO. Primary cells (peritoneal macrophage cells) were derived from mice of both sexes (equal distribution). According to the Research-SAGER-guidelines, immortalized cell lines (HEK293T, RAW264.7, Cul3-/- and Immt-/-) are not required to state the sex.
Authentication	The cell lines purchased from ATCC have been authenticated.
Mycoplasma contamination	We confirm that all cell lines tested negative for mycoplasma contamination.
Commonly misidentified lines (See ICLAC register)	There are no commonly misidentified lines in the study.

Animals and other research organisms

Policy information about studies involving animals; ARRIVE guidelines recommended for reporting animal research, and Sex and Gender in Research

Laboratory animals	Specified Pathogen Free (SPF) sex-matched BALB/c mice at approximately 6-8 weeks (Vital River Laboratory Animal Technology Co., Ltd) were maintained under barrier conditions in a BSL-3 biohazard animal room at 25-27℃ and provided with free water and diet and a 12 h light/dark cycle. At the beginning of experiment, mice were randomly grouped and treated accordingly in each experimental condition.
Wild animals	We state that the study did not involve wild animals.
Reporting on sex	Sex-matched BALB/c mice were considered in the study.
Field-collected samples	This study did not involve samples collected from the field.
Ethics oversight	All animal experiments were approved by the Laboratory Animal Welfare and Ethics Committee of the Third Military Medical University (AMUWEC20223354)

Note that full information on the approval of the study protocol must also be provided in the manuscript.

Flow Cytometry

Plots

Confirm that:

☒ The axis labels state the marker and fluorochrome used (e.g. CD4-FITC).

☒ The axis scales are clearly visible. Include numbers along axes only for bottom left plot of group (a 'group' is an analysis of identical markers).

☒ All plots are contour plots with outliers or pseudocolor plots.

☒ A numerical value for number of cells or percentage (with statistics) is provided.

Methodology

Sample preparation	To detect the mtROS in RAW264.7 cells or mouse PMs after B. pseudomallei infection, cells were infected with B. pseudomallei at the indicated MOI for indicated time points. Cells were washed with PBS for three times, stained by 5 µM mitoSOX (Thermo Fisher, M36008) for 15 min at 37℃ in dark place, washed with PBS for three times, and then collected with PBS in the sample tubes of flow cytometry according to the manufacturer's instructions. To detect the MMP in RAW264.7 cells or mouse PMs, cells were infected with B. pseudomallei as described above. Cells were washed with PBS for three times, stained by 100 nM TMRM (Invitrogen, T668) for 30 min at 37℃ in dark place, washed with PBS for three times, and then collected with PBS in the sample tubes of flow cytometry
Instrument	The MFI data were acquired by the Fortessa flow cytometer (BD Biosciences)
Software	Data was analyzed by FlowJo software v10.
Cell population abundance	Approximately 10,000 cells
Gating strategy	The Mean Fluorescence Intensity (MFI) of the untreated group is used as the baseline, where the MFI below the baseline is

Gating strategy

negative, and above the baseline is positive.

☐ Tick this box to confirm that a figure exemplifying the gating strategy is provided in the Supplementary Information.
