# Supplementary material for: Burkholderia pseudomallei BipD modulates host mitophagy to evade killing
Source: Nat Commun. 2024 Jun 4;15:4740. doi: 10.1038/s41467-024-48824-x (PMC11150414; doi:10.1038/s41467-024-48824-x)
Supplement: Supplementary file 9 — Source Data [file 41467_2024_48824_MOESM9_ESM.zip › 04 source data for uncropped blots and gels.pdf]

Figure 1c

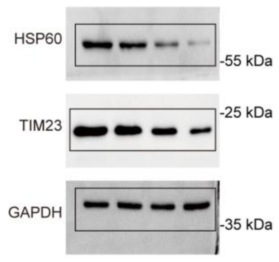

Figure 1d

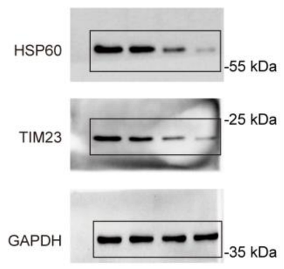

Figure 1l

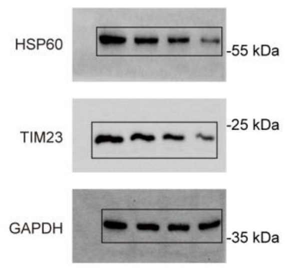

Figure 3b

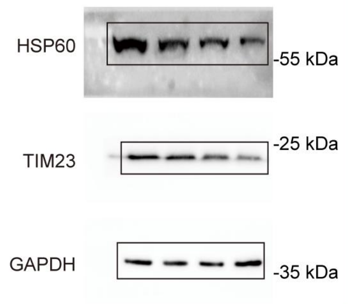

Figure 3k

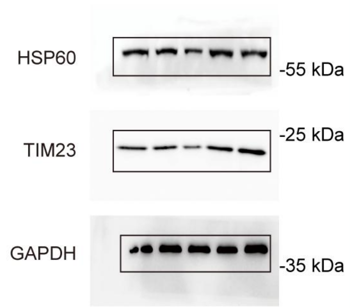

Figure 3b

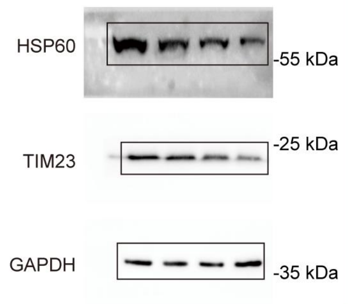

Figure 3k

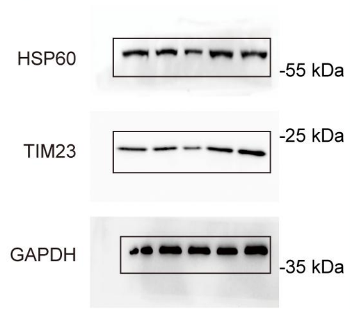

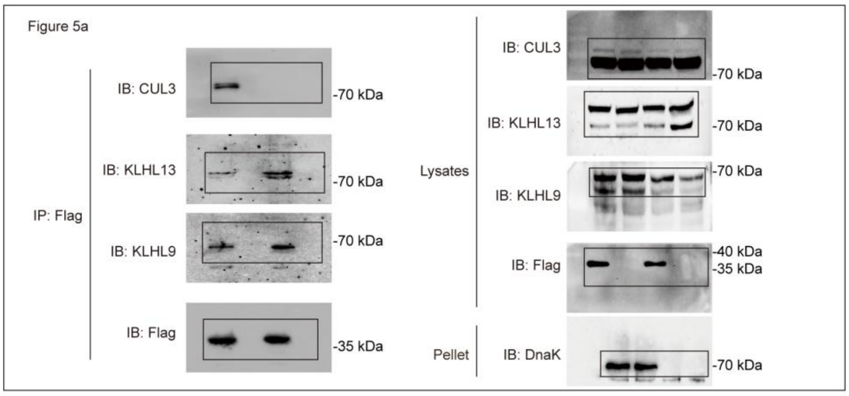
Figure 5a
IP: Flag
IB: CUL3
-70 kDa
IB: KLHL13
-70 kDa
IB: KLHL9
-70 kDa
IB: Flag
-35 kDa
Lysates
IB: CUL3
-70 kDa
IB: KLHL13
-70 kDa
IB: KLHL9
-70 kDa
IB: Flag
-40 kDa
-35 kDa
Pellet
IB: DnaK
-70 kDa

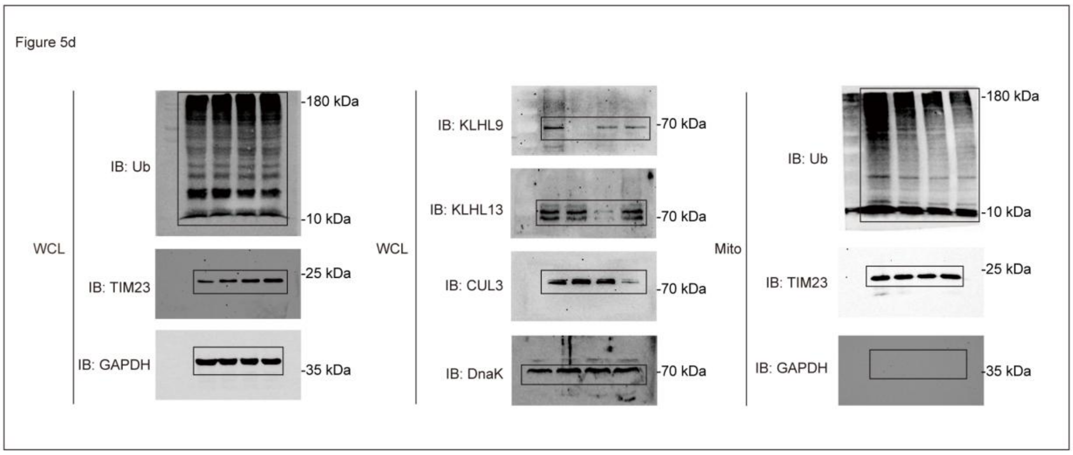
Figure 5d
WCL
IB: Ub
-180 kDa
-10 kDa
IB: TIM23
-25 kDa
IB: GAPDH
-35 kDa
WCL
IB: KLHL9
-70 kDa
IB: KLHL13
-70 kDa
IB: CUL3
-70 kDa
IB: DnaK
-70 kDa
Mito
IB: Ub
-180 kDa
-10 kDa
IB: TIM23
-25 kDa
IB: GAPDH
-35 kDa

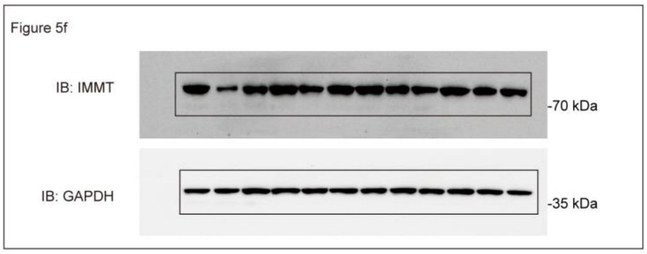
Figure 5f
IB: IMMT
-70 kDa
IB: GAPDH
-35 kDa

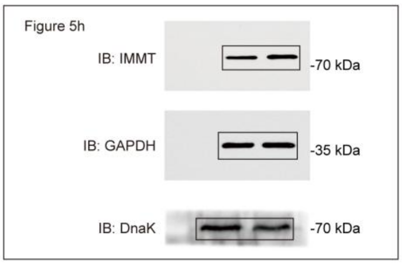
Figure 5h
IB: IMMT
-70 kDa
IB: GAPDH
-35 kDa
IB: DnaK
-70 kDa

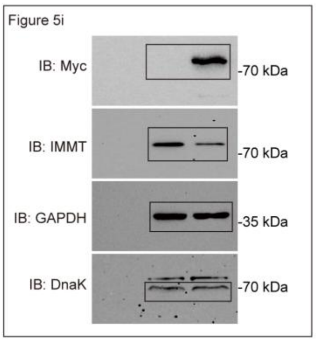
Figure 5i
IB: Myc
-70 kDa
IB: IMMT
-70 kDa
IB: GAPDH
-35 kDa
IB: DnaK
-70 kDa

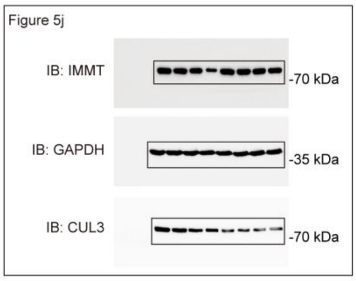
Figure 5j
IB: IMMT
-70 kDa
IB: GAPDH
-35 kDa
IB: CUL3
-70 kDa

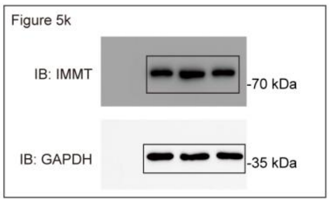
Figure 5k
IB: IMMT
-70 kDa
IB: GAPDH
-35 kDa

Figure 6a

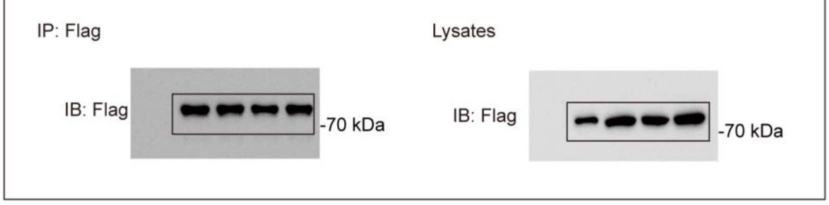

Figure 6b

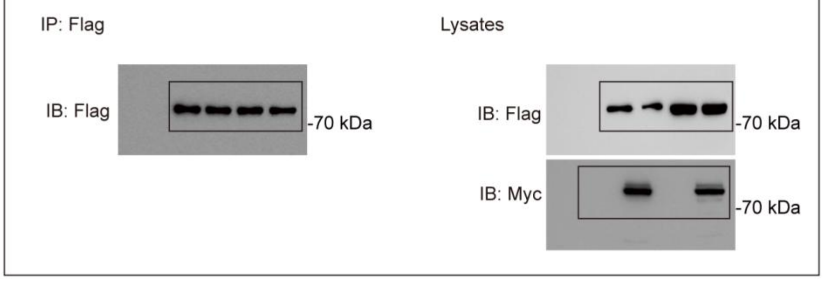

Figure 6d

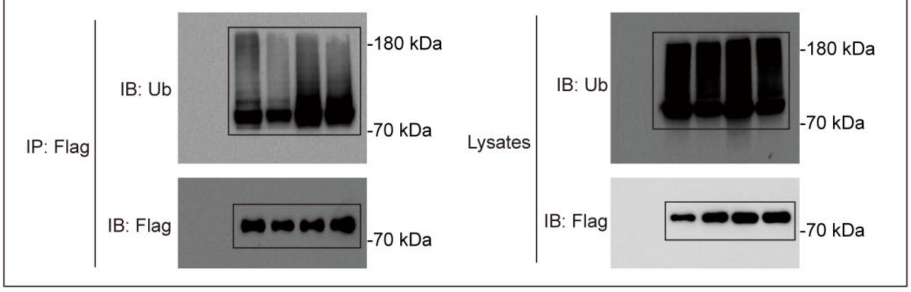

Figure 6f

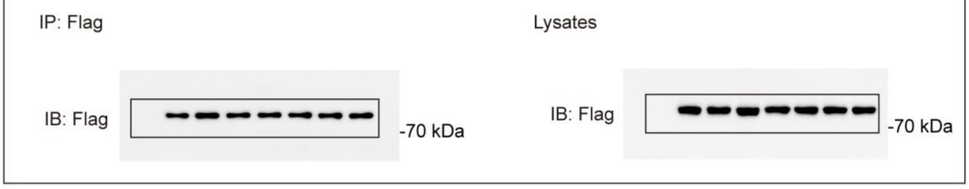

Figure 7b

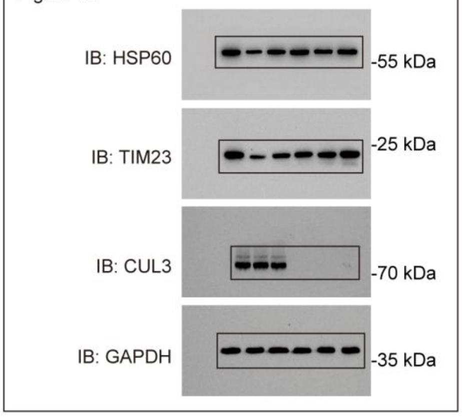

Figure 7d

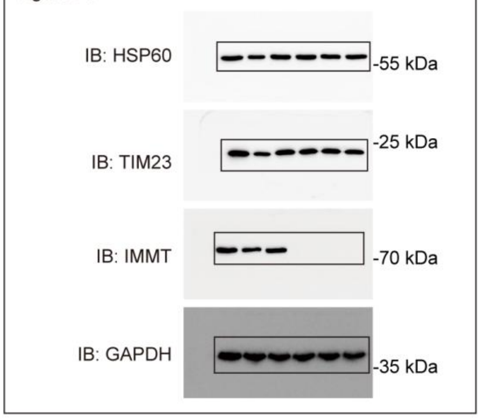

Figure S1d

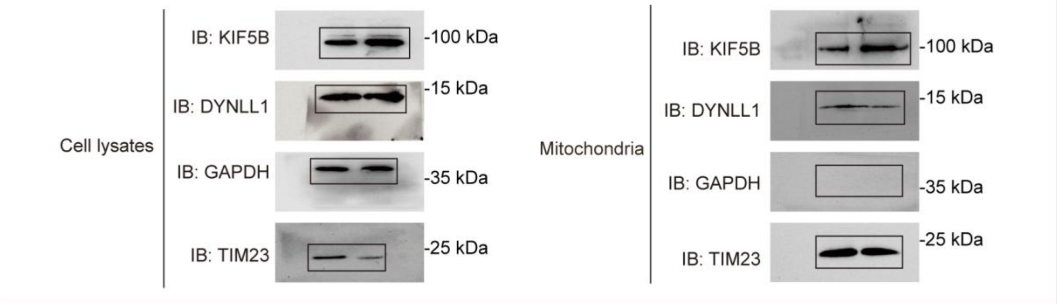

Figure S2b

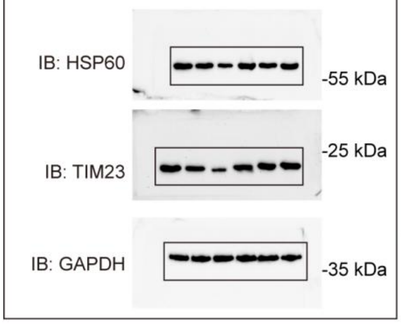

Figure S2d

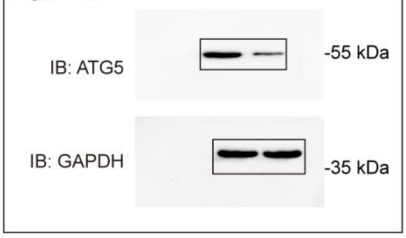

Figure S2g

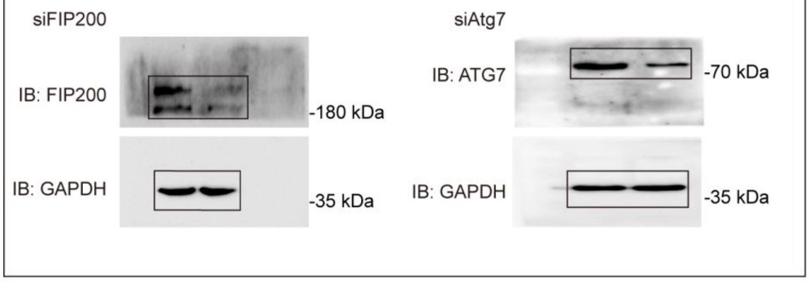

Figure S3a

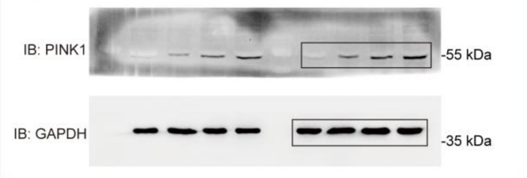

Figure S3b

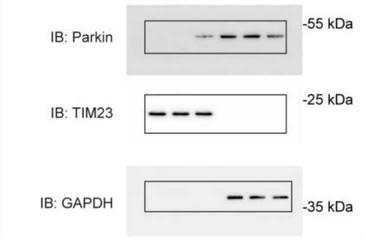

Figure S3e

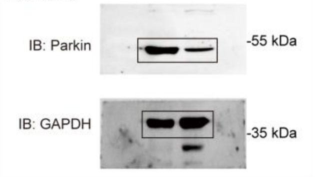

Figure S3f

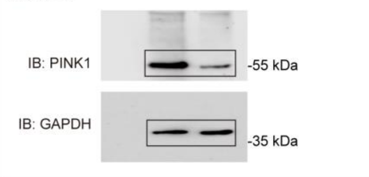

Figure S3k

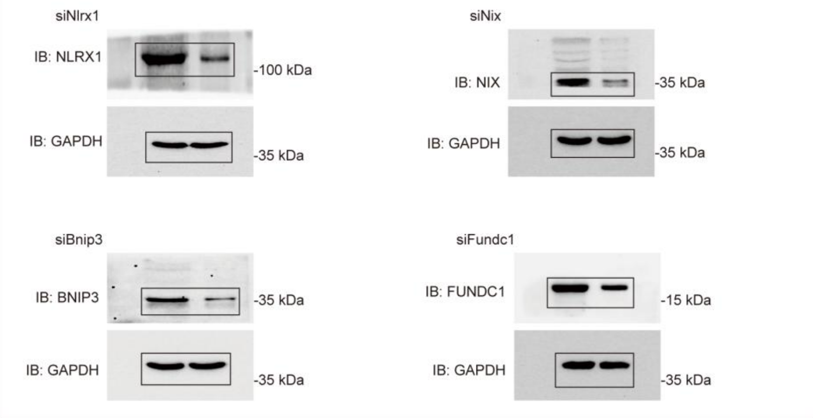

Figure S5b

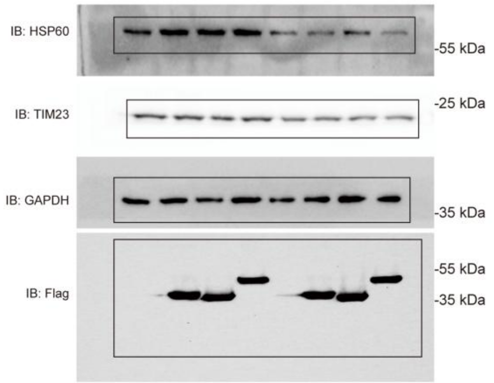

Figure S5c

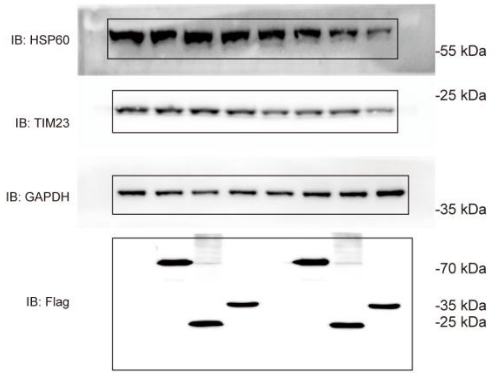

Figure S5d

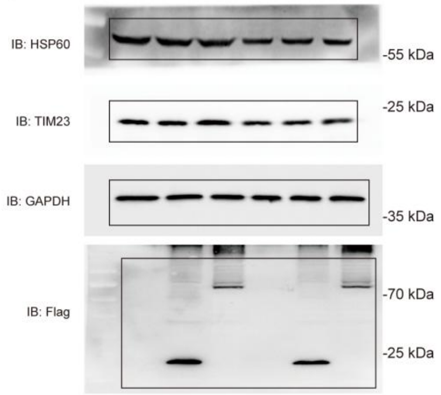

Figure S5f

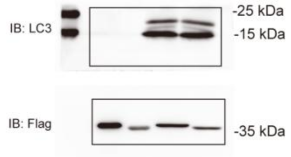

Figure S6b

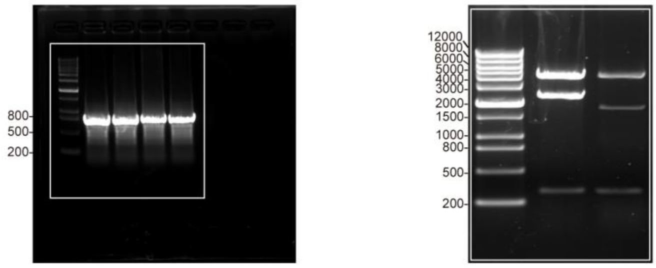

Figure S7b

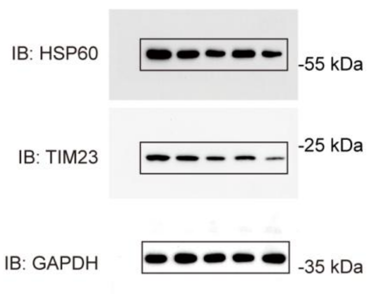

Figure S7e

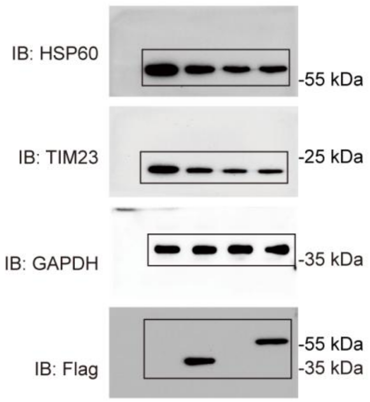

Figure S8a

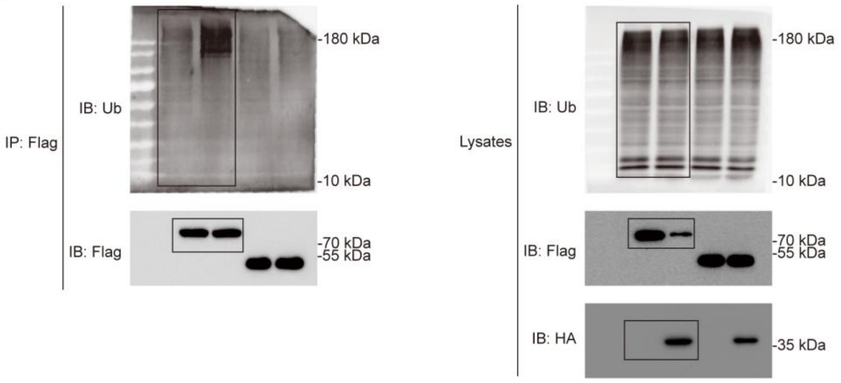

Figure S8b

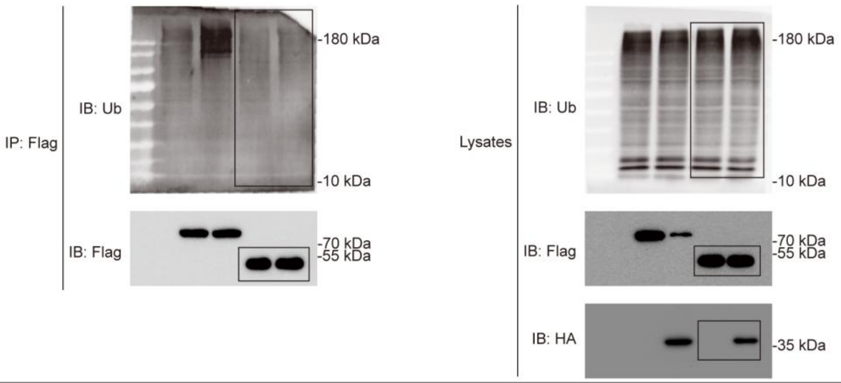

Figure S9d

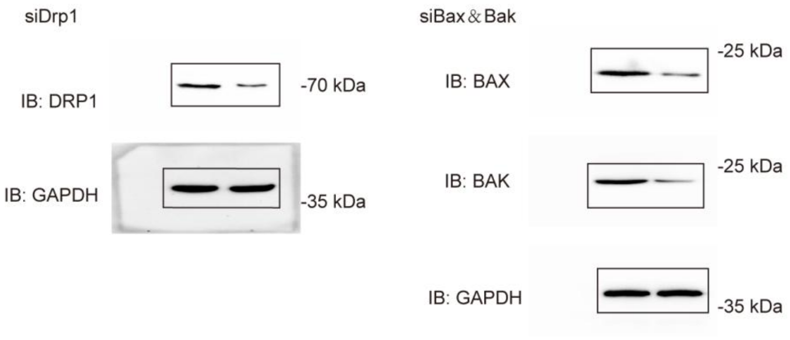
siDrp1
IB: DRP1
-70 kDa
IB: GAPDH
-35 kDa
siBax＆Bak
IB: BAX
-25 kDa
IB: BAK
-25 kDa
IB: GAPDH
-35 kDa
